# Mechanism of allosteric inhibition of HIV-1 reverse transcriptase revealed by single-molecule and ensemble fluorescence

**DOI:** 10.1093/nar/gku819

**Published:** 2014-09-17

**Authors:** Grant D. Schauer, Kelly D. Huber, Sanford H. Leuba, Nicolas Sluis-Cremer

**Affiliations:** 1 Program in Molecular Biophysics and Structural Biology, University of Pittsburgh School of Medicine, Hillman Cancer Center, 5117 Centre Avenue, Pittsburgh, PA 15213, USA; 2 Department of Cell Biology, University of Pittsburgh School of Medicine, Hillman Cancer Center, 5117 Centre Avenue, Pittsburgh, PA 15213, USA; 3 Department of Medicine, Division of Infectious Diseases, University of Pittsburgh School of Medicine, Scaife Hall, 3550 Terrace Street, Pittsburgh, PA 15261, USA

## Abstract

Non-nucleoside reverse transcriptase (RT) inhibitors (NNRTIs) are routinely used to treat HIV-1 infection, yet their mechanism of action remains unclear despite intensive investigation. In this study, we developed complementary single-molecule fluorescence and ensemble fluorescence anisotropy approaches to discover how NNRTIs modulate the intra-molecular conformational changes and inter-molecular dynamics of RT-template/primer (T/P) and RT–T/P–dNTP complexes. We found that NNRTI binding to RT induces opening of the fingers and thumb subdomains, which increases the dynamic sliding motion of the enzyme on the T/P and reduces dNTP binding affinity. Further, efavirenz promotes formation of the E138-K101 salt bridge between the p51 and p66 subunits of RT, which contributes to opening of the thumb/fingers subdomains. Engineering a more polar salt bridge between p51 and p66 resulted in even greater increases in the thumb/fingers opening, RT sliding, dNTP binding disruption and *in vitro* and *in vivo* RT inhibition than were observed with wild-type RT. We also observed that K103N, a clinically relevant NNRTI resistance mutation, does not prevent binding between efavirenz and RT-T/P but instead allows formation of a stable and productive RT–T/P–dNTP complex, possibly through disruption of the E138-K101 salt bridge. Collectively, these data describe unique structure–activity–resistance relationships that could be exploited for drug development.

## INTRODUCTION

The multifunctional enzyme HIV-1 reverse transcriptase (RT) catalyzes the conversion of viral single-stranded RNA into double-stranded DNA that encodes the HIV-1 genome and is subsequently integrated into the host genome. RT is an asymmetric heterodimer composed of a 66-kDa (p66) subunit and a p66-derived 51-kDa (p51) subunit (1). The polymerase domain in p66 displays an overall architectural similarity to the Klenow fragment of *Escherichia coli* and is divided into fingers, palm and thumb subdomains (2). The p66 subunit also contains a connection and an RNase H domain (3). The p51 subunit is composed of the polymerase and connection domains, although their spatial arrangement differs markedly from p66. The p66 subunit adopts an open, catalytically competent conformation that can accommodate the template/primer (T/P) substrate, whereas p51 is in a closed conformation and plays a largely structural role (4).

The non-nucleoside RT inhibitors (NNRTIs), such as efavirenz (EFV), nevirapine (NVP) and rilpivirine (RPV), bind to a hydrophobic pocket in the palm subdomain of p66 that is ∼10 Å from the polymerase active site, inhibiting reverse transcription via an allosteric mechanism of action (5,6). This pocket does not exist in the absence of an inhibitor; rather, drug binding causes the side chains of Y181 and Y188 to flip from a ‘down’ to an ‘up’ orientation, generating the NNRTI-binding pocket (7–9). In addition, NNTRIs force the p66 thumb into an open, extended position, which led to the hypothesis that NNRTIs induce ‘molecular arthritis’, whereby the relative domain movements in RT, thought to be necessary for the catalytic cycle of the enzyme, are inhibited (3).

NNRTI binding also alters key structural elements in the polymerase active site, including the YMDD motif, which coordinates the divalent metal ions required for phosphodiester bond formation, and the ‘primer grip’, which positions the 3′-OH end of DNA primer for catalysis (7–9). Further, the deoxynucleotide triphosphate (dNTP) binding pocket is distorted in the crystal structure of RT bound to T/P and NVP, which led to the suggestion that this complex cannot bind dNTP (10). However, transient kinetic analyses have shown that NNRTI binding to RT does not prevent the formation of the RT–T/P–dNTP complex but instead significantly slows the rate of nucleotide incorporation (6,11). Collectively, these studies highlight a disconnect between the available kinetic and structural studies focused on NNRTI mechanism of action. Characterization of such structure–activity relationships would considerably aid in the rational design of more effective inhibitors.

RT exists in multiple mechanistic forms, including free enzyme, an RT–T/P (binary) complex and an RT–T/P–dNTP (ternary) complex. Even with a rich catalog of RT-NNRTI structures, we still do not know how NNRTIs alter the intra-molecular conformational changes or the inter-molecular dynamics of binary and ternary complexes, the functionally relevant forms of RT. Previous studies examining intermolecular single-pair Förster resonance energy transfer (spFRET) established that RT can slide and flip on T/P substrates and that NNRTIs increase the sliding of RT on T/P substrates (12,13), suggesting that the thumb/fingers grip on the substrate plays a role in RT inhibition; however, these pioneering studies did not provide experimental evidence for the conformational changes within RT that are associated with these dynamic intermolecular changes.

In this study, we developed novel single-molecule and ensemble biophysical assays to characterize the relationship between NNRTI-induced changes in inter-molecular dynamics and intra-molecular changes within RT. We provide the first report of conformational changes associated with RT in complex with T/P, NNRTI and dNTP that, taken together, indicate a unique molecular mechanism of allostery through which the inhibitors prevent reverse transcription by modulating the dynamic interplay among RT conformation, RT sliding and dNTP binding. We further demonstrate that K103N, a clinically observed mutant that confers viral resistance to EFV, does not affect NNRTI binding but instead directly targets and prevents this allosteric mechanism.

## MATERIALS AND METHODS

### RT constructs

The wild type (WT) and mutant HIV-1 RT enzymes used in the single-molecule protein-induced fluorescence enhancement (PIFE) and anisotropy assays were expressed from the p6HRT-Prot expression vector and purified as described previously (14,15). The RT constructs used for the single-pair FRET assays were expressed from the pETDuet-1 vector and purified as described previously (16).

### Fluorescence anisotropy

Fluorescence anisotropy experiments were performed as previously described (17,18). Assays were carried out using a fluorescein-labeled T/P substrate. The concentration of T/P in all experiments was 5 nM (in a 400-μl cuvette). Data were analyzed as described in the Supplementary Materials and Methods section.

### Single-molecule total internal reflection fluorescence microscopy

Single-molecule total internal reflection fluorescence (TIRF) microscopy was performed as described previously (19). For the PIFE assays, Cy3-labeled T/P molecules were surface-tethered to a polyethylene glycol (PEG) treated flow cell via a biotin:streptavidin:biotin-PEG linkage. The concentrations of T/P were typically 20 pM, which provided an optimal surface density of ∼100 molecules per field of view. The flow cell was introduced with 250 nM RT and incubated for 5 min, followed extensive washing to remove unbound protein. For the FRET assays, unlabeled T/P was surface-tethered by introducing 2 μM T/P into the flow cell to allow formation of a surface-dense layer of T/P substrates. Labeled RT (40 pM) was then introduced into the flow cell and allowed to bind to the T/P. Unbound RT was removed by washing. The PIFE and FRET data were analyzed as described in Supplementary Materials and Methods.

### Accelerated molecular dynamics simulations of PIFE

Accelerated molecular dynamics (AMD) simulations were performed on the atomic coordinates of the ternary RT-T/P-dTTP complex (PDB: 1RTD) (20) modified at the 5′-end of the primer with Cy3 and included harmonic constraints on all Cα and P atoms not within 25 Å of Cy3. Each RT-Cy3 distance variation was sampled for 50 ns in triplicate. Full simulation conditions are described in Supplementary Materials and Methods.

### HIV-1 drug susceptibility assays

The genes for K103N, E138D/K101R and K101R/K103N/E138D RT were cloned into HIV-1_LAI_ (11). NNRTI susceptibility was determined in TZM-bl cells as described previously (21).

### Activity of RT constructs

The RNA- and DNA-dependent DNA polymerase activities of the purified and labeled RTs were assessed as described previously (14). The concentration of NNRTI required to inhibit 50% of the RT DNA polymerase activity (IC_50_) was determined as described previously (14).

## RESULTS

### Fluorescence anisotropy captures the sliding distributions of RT on the T/P substrate

**Figure 1.**
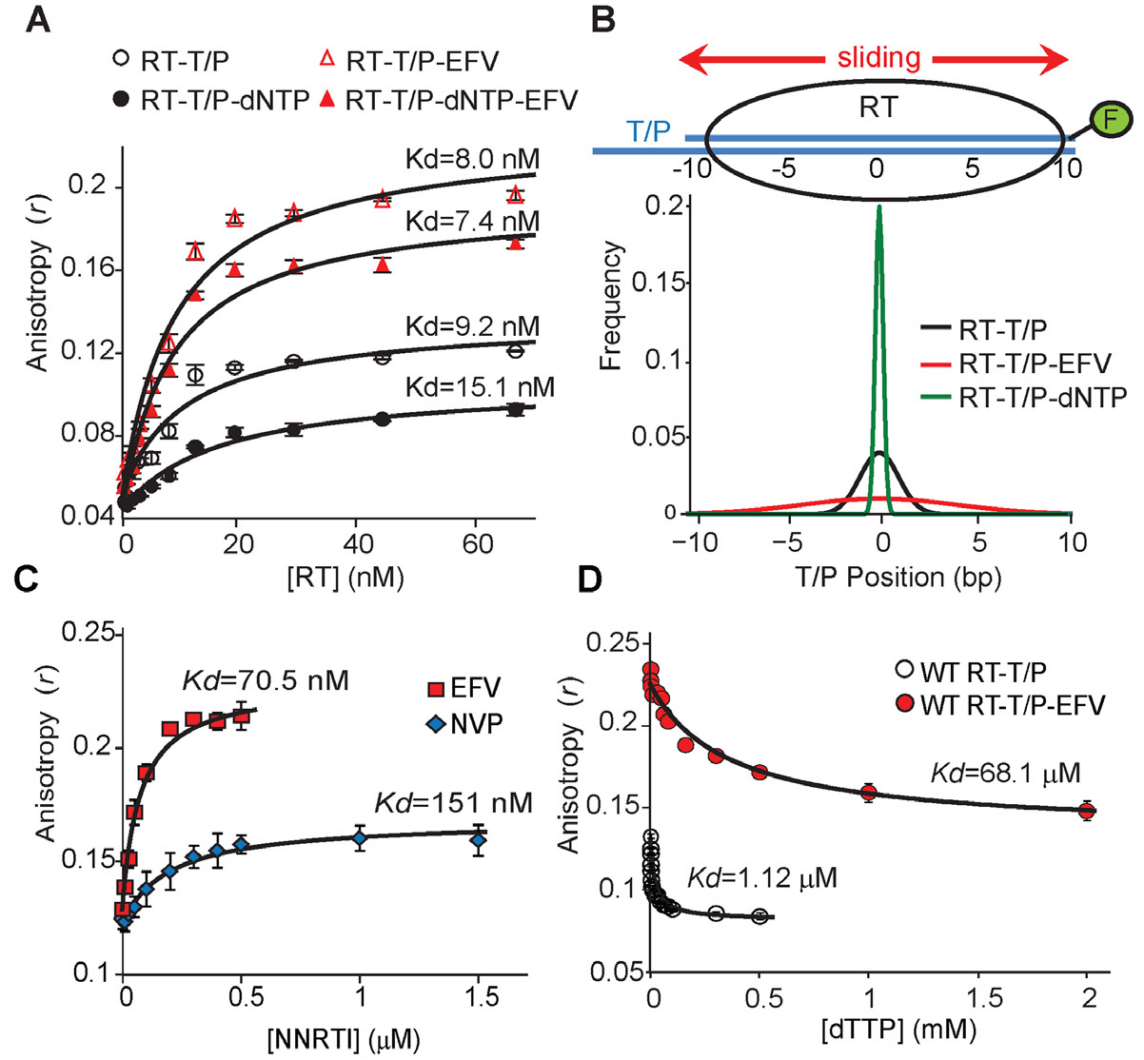
Interactions between RT and T/P measured by fluorescence anisotropy. (**A**) Binding isotherm for RT to T/P in the absence or presence of EFV and/or dNTP. Data are reported as mean ± SEM from at least three separate experiments. (**B**) Model-derived distributions of the RT–T/P, RT–T/P–EFV and RT–T/P–dNTP complexes. (**C**) NVP and EFV binding to the WT RT–T/P complex measured by anisotropy. (**D**) dTTP binding to WT RT–T/P complexes in the absence and presence of EFV measured by anisotropy. Data reported as mean ± SEM.

Our primary goal was to develop single-molecule assays to assess the intra-molecular conformational changes of RT and the inter-molecular dynamics of the RT–T/P and RT–T/P–dNTP complexes. We first evaluated the thermodynamic stability of these complexes by measuring the changes in the anisotropy (*r*) of a fluorescein dye attached to the 5′ end of the DNA primer (Figure 1A). The T/P duplex used in these and all other experiments herein was identical in sequence to the substrate reported in a crystal structure of the RT–T/P–dNTP ternary complex (20) and was chain-terminated with 2′,3′-dideoxycytosine-monophosphate to allow capture of the stable ternary complex. We found that RT binding to the T/P resulted in an increase in *r*, which allowed us to calculate a dissociation constant (*K*_d_) of 9.2 ± 1.0 nM for the RT–T/P complex. Surprisingly, the addition of the next correct dNTP (dTTP) and/or EFV did not change the *K*_d_ of the RT–T/P interaction but significantly affected the maximum *r* value under saturating RT concentrations (Figure 1A); additionally, our observation that the *r* maxima were increased by EFV addition and decreased by dTTP addition was opposite of the trend we predicted.

Physical modeling of these data (Supplementary Figure S1A and B) revealed that the experimentally observed differences in *r* maxima did not result from changes in the effective hydrodynamic radius of RT–T/P in complex with dTTP and/or EFV but rather could be attributed to changes in the mobility of RT on the T/P substrate and its altered interaction with its terminal fluorescein (Supplementary Figure S1). Since prior spFRET studies revealed that RT slides on the T/P substrate (12), we considered a coarse-grained physical model in which RT interacts with the fluorescein dye as it slides along the T/P (Supplementary Figure S1C). From this model, we were able to predict normal distributions for the RT–T/P, RT–T/P–dNTP and RT–T/P–EFV complexes (Figure 1B; Supplementary Figure S1D). This analysis revealed that RT has a tight distribution on the T/P substrate in the presence of dNTP, which is indicative of a stable RT–T/P–dNTP complex, but a much broader distribution in the presence of EFV, which reflects RT sliding on the T/P substrate. Therefore, the observed anisotropy value in our assays can be directly related to the mobility of RT on its T/P substrate, where high *r* values indicate high sliding mobility and vice versa.

### Determination of NNRTI or dNTP binding to the RT–T/P complex by anisotropy

The increase in the maximum *r* value between the RT–T/P and RT–T/P–EFV complexes (Figure 1A) allowed us to quantify NVP, EFV and RPV binding to the RT–T/P complex (Figure 1C; Supplementary Figure S2A and B), while the decrease in maximum *r* value between the RT–T/P and RT–T/P–dNTP complexes allowed us to quantify dNTP binding (Figure 1D; Supplementary Figure S2C). As expected, EFV bound with greater affinity (*K*_d_ = 70.5 nM) than did NVP (*K*_d_ = 151 nM). Under saturating inhibitor concentrations, EFV binding to the RT–T/P complex also resulted in a larger increase in *r* (Figure 1C), which indicates that, compared with NVP, EFV induces more sliding of RT on the T/P. Consistent with this finding, the RT–T/P–EFV complex bound dTTP with significantly lower affinity (Figure 1D) than did NVP (Supplementary Figure S2C). RPV bound with similar affinity (82.5 nM) as EFV, resulting in a comparable increase in *r* value (Supplementary Figure S2A and B). These data demonstrate the differential binding affinity of NNRTI and the resultant distribution of RT on the T/P, as well as the inverse relationship between the binding affinity of NNRTI and the ability of the NNRTI–RT–T/P complex to bind dNTP.

### K103N allows EFV-bound RT to form a polymerase-competent complex

**Figure 2.**
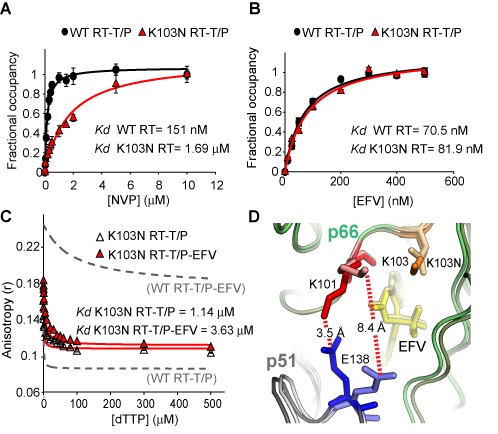
K103N allows the EFV-bound enzyme to form a stable polymerase-competent RT–T/P–dNTP complex. (**A**) NVP or (**B**) EFV binding to the WT and K103N RT–T/P complexes measured by fluorescence anisotropy. Data are reported as mean ± SEM. (**C**) dTTP binding to the K103N RT–T/P complex in the absence and presence of EFV. Data are reported as mean ± SEM. Dashed lines represent analogous dTTP titrations to WT RT (Figure 1D) for direct comparison. (**D**) Overlay of WT (1FK9) and K103N (1FKO) RT in complex with EFV. K103N disrupts the salt bridge between K101 and E138.

The K103N substitution in RT is the most commonly reported resistance mutation arising following clinical use of EFV. When we quantified NVP and EFV binding to K103N RT, we found that while the K103N substitution decreased NVP binding affinity by ∼10-fold (Figure 2A), it surprisingly had no effect on EFV binding (Figure 2B). Of note, the K103N substitution did not decrease the binding affinity of RPV for the RT–T/P complex (Supplementary Figure S2A and B), an observation which is consistent with the observation that RPV retains activity against this resistance mutation (22). Furthermore, we found that the binding isotherms for dTTP to the K103N RT–T/P complex were largely similar in the absence and presence of EFV: the *K*_d_ value for dTTP binding was only increased 3-fold in the presence of inhibitor (Figure 2C). These data suggest that K103N does not prevent EFV from binding to the RT–T/P complex but instead allows the inhibitor-bound enzyme to form a stable polymerase-competent ternary complex.

### E138-K101 salt bridge between the p51 and p66 subunits of RT associated with EFV binding and inhibitory activity

In the RT-EFV crystal structure (23), direct contact between EFV and K103 is not observed, and a stereochemical basis for the loss of NNRTI binding due to K103N cannot be inferred from these structures (24). However, we observed that K103 in p66 appears to ideally position K101 to form a salt bridge with E138 in p51 (Figure 2D). In the crystal structure of K103N RT in complex with EFV solved by Ren *et al.* (23), the K103N substitution was found to disrupt the electrostatic repulsion of K101, and consequently eliminated the salt bridge between K101 and E138 (Figure 2D and Supplementary Movie 1). However, in the K103N RT-EFV structure solved by Lindberg *et al.* (25), K103N did not appear to disrupt the K101-E138 salt bridge. Interestingly, previous elastic network model and molecular dynamics simulations have suggested that the NNRTI-binding pocket is proximal to an important hinge site that controls RT fingers and thumb dynamics (26,27). These dynamics could, in turn, alter the distribution of RT on the T/P, as first hypothesized by Liu *et al.* (12). Notably, residues 87–110 in p66 and 134–141 in p51 were reported to be important motifs in this hinge site (26). In light of both the available crystal structure and molecular dynamics data, we hypothesized that that the E138-K101 salt bridge may play an important role in the inhibitory activity of EFV toward WT and K103N RT.

**Figure 3.**
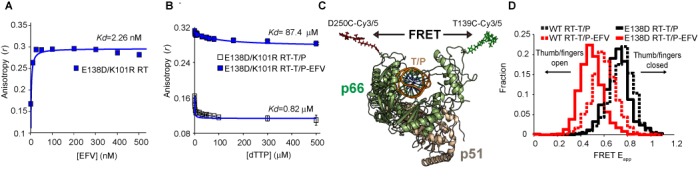
E138-K101 salt bridge increases RT dynamics on the T/P by modulating the thumb/fingers distance in RT–T/P complexes. (**A**) EFV binding to the E138D RT–T/P complex measured by fluorescence anisotropy. (**B**) dTTP binding to the K103N RT–T/P complex in the absence and presence of EFV. Data are reported as mean ± SEM. (**C**) Schematic of FRET construct, indicating the distances measured. Energy transfer is measured between Cy3 and Cy5 dyes attached to the fingers (residue 139) and thumb (residue 250) subdomains in the p66 subunit of RT. (**D**) FRET histograms for the WT RT–T/P complex in the absence (*N* = 222 traces) or presence of EFV, and for the E138D RT–T/P complex in the absence (*N* = 129) or presence of EFV (*N* = 94).

We therefore manipulated this electrostatic interaction, engineering a stronger salt bridge by increasing the polarity of the side chains through the introduction of arginine (K101R; p*K*_a_ change from 10.53 to 12.48) and aspartic acid (E138D; p*K*_a_ change from 4.25 to 3.65) (28), and evaluated the effect of these substitutions on RT inhibition and virus replication (see the Materials and Methods section). The E138D/K101R mutation increased viral susceptibility (as measured by IC_50_ and EC_50_) to EFV by up to 7-fold, which appeared to be primarily driven by the E138D mutation (Table 1). Interestingly, the E138D/K101R mutations were found to partially compensate for EFV resistance due to K103N (Table 1). This finding provides support for our hypothesis that the K101-E138 salt bridge partially contributes toward the inhibitory activity of EFV toward WT and K103N HIV-1. When we used anisotropy to assess binding to the E138D/K101R RT–T/P complex, we found that EFV bound with increased affinity and induced an even larger change in the maximum *r* value at saturating inhibitor concentrations (Figure 3A). Furthermore, the E138D/K101R EFV–RT–T/P complex bound dTTP with dramatically reduced affinity (Figure 3B). Collectively, these data suggest that a salt bridge between K101 and E138 contributes to the inhibitory activity of EFV.

**Table 1.** Susceptibility of WT and mutant HIV-1 RT or virus to NNRTIs

	Inhibition of					
Genotype of RT or virus	RT RNA-dependent DNA polymerase activity			HIV-1 replication in P4/R5 cells		
	IC_50_ (nM)^a^	Fold-R^b^	*P* value	EC_50_ (nM)^c^	Fold-R^d^	*P* value
**Efavirenz**						
WT	47.6 ± 1.1	-	-	1.3 ± 0.2	-	-
E138D	17.6 ± 2.1	0.4	0.003	-	-	-
K101R	37.4 ± 1.8	0.8	N.S.	-	-	-
K101R/E138D	7.5 ± 2.4	0.2	< 0.001	0.2 ± 0.0	0.2	0.005
K103N	> 1000	> 1000	0	64.0 ± 18.7	49.2	0.014
K101R/E138D/K103N	-	-	-	15.9 ± 2.4	12.2	< 0.001
T139C/D250C	49.9 ± 12.9	1.1	N.S.	-	-	-
T139C/D250C/K103N	4566.7 ± 115.5	95.9	< 0.001	-		
T139C/D250C/E138D	14.3 ± 4.0	0.3	0.001	-	-	-
**Nevirapine**						
WT	2323.6 ± 179.2	-	-	24.6 ± 5.9	-	-
E138D	2176 ± 68.5	0.9	N.S.			
K101R	2936 ± 599.3	1.3	N.S.			
K101R/E138D	2102 ± 290.7	0.9	N.S.	21.6 ± 16.8	0.9	N.S.
K103N				> 200	> 10	0
K101R/E138D/K103N				> 200	> 10	0
**Rilpivirine**						
WT	22.5 ± 2.2	-	-	0.37 ± 0.06	-	-
E138D	8.3 ± 1.2	0.4	0.4	-	-	-
K101R	15.5 ± 7.6	0.7	0.003	-	-	-
K101R/E138D	11.2 ± 0.8	0.5	<0.001	0.06 ± 0.01	0.2	<0.001
K103N				0.4 ± 0.1	1.1	N.S.
K101R/E138D/K103N				0.12 ± 0.03	0.3	0.002

^a^Concentration of NNRTI required to inhibit 50% of the *in vitro* RNA-dependent DNA polymerase activity of RT. Data are the mean ± SD from at least four different experiments.

^b^Fold resistance defined as IC_50_^mutant^/IC_50_^WT^.

^c^Concentration of NNRTI required to inhibit 50% of HIV-1 replication in TZM-bl cells. Data are the mean ± SD from at least three different experiments.

^d^Fold resistance defined as EC_50_^mutant^/EC_50_^WT^.

### E138-K101 salt bridge modulates the thumb/fingers distance in RT–T/P complexes

Next, we used spFRET to measure conformational changes in the fingers and thumb in the p66 subunit of WT RT–T/P and RT–T/P–dNTP complexes in the absence and presence of EFV or NVP. Briefly, we generated a FRET RT construct that allowed us to site-specifically label the fingers (T139C) and thumb (D250C) of p66 with Cy3 and Cy5 dyes (Figure 3C). The construction, purification and characterization of FRET RT is described in the Materials and Methods section, and the activity of all mutant and chemically modified RTs is shown in Supplementary Figure S3. The Cy3/5-labeled RT was then infused into a PEGylated flow cell containing a surface-dense layer of unlabeled, surface-tethered T/P. After allowing RT to bind T/P, unbound protein was removed by washing, and the apparent energy transfer (*E*_app_) between Cy3 and Cy5 in individual complexes was monitored using TIRF microscopy (Supplementary Figure S4). Cy3/5-labeled RT did not bind to PEGylated flow cells lacking DNA substrates, indicating that the observed FRET signals originated exclusively from T/P-bound RT molecules (Supplementary Figure S5).

In the WT RT–T/P complex, *E*_app_ was centered at 0.74 (Figure 3D). In the presence of NVP and EFV, *E*_app_ decreased to 0.68 and 0.57, respectively, indicating that the fingers and thumb opened up by an average of 2.0 and 5.9 Å, respectively (Figure 3D; Supplementary Figure S6A). We next generated stable RT–T/P–dNTP complexes and infused NVP or EFV in the flow cell (Supplementary Figure S6B). EFV increased the distance between fingers and thumb in RT–T/P–dNTP. However, NVP did not affect the FRET histogram for WT RT–T/P–dNTP, suggesting that this inhibitor may not bind to the ternary complex, consistent with what was previously proposed (29).

Next, we introduced E138D into the p51 subunit of the FRET RT construct to assess the effect of EFV binding on RT conformation. We found that the center of the *E*_app_ distribution decreased from 0.69 for the E138D RT–T/P complex to 0.46 when EFV was added (Figure 3D; Supplementary Table S1). This resulted in the fingers and thumb moving apart by ∼8.4 Å, which was significantly larger than the distance observed for the WT RT–T/P complex in the presence of EFV or NVP (Figure 3D; Supplementary Figure S6D and Supplementary Table S1). These data suggest an inverse relationship between the binding affinity of the NNRTI and the distance between the fingers and thumb in the NNRTI–RT–T/P complex for EFV and NVP.

### K103N eliminates EFV-induced RT sliding and allows EFV-bound RT to form a polymerase competent complex

**Figure 4.**
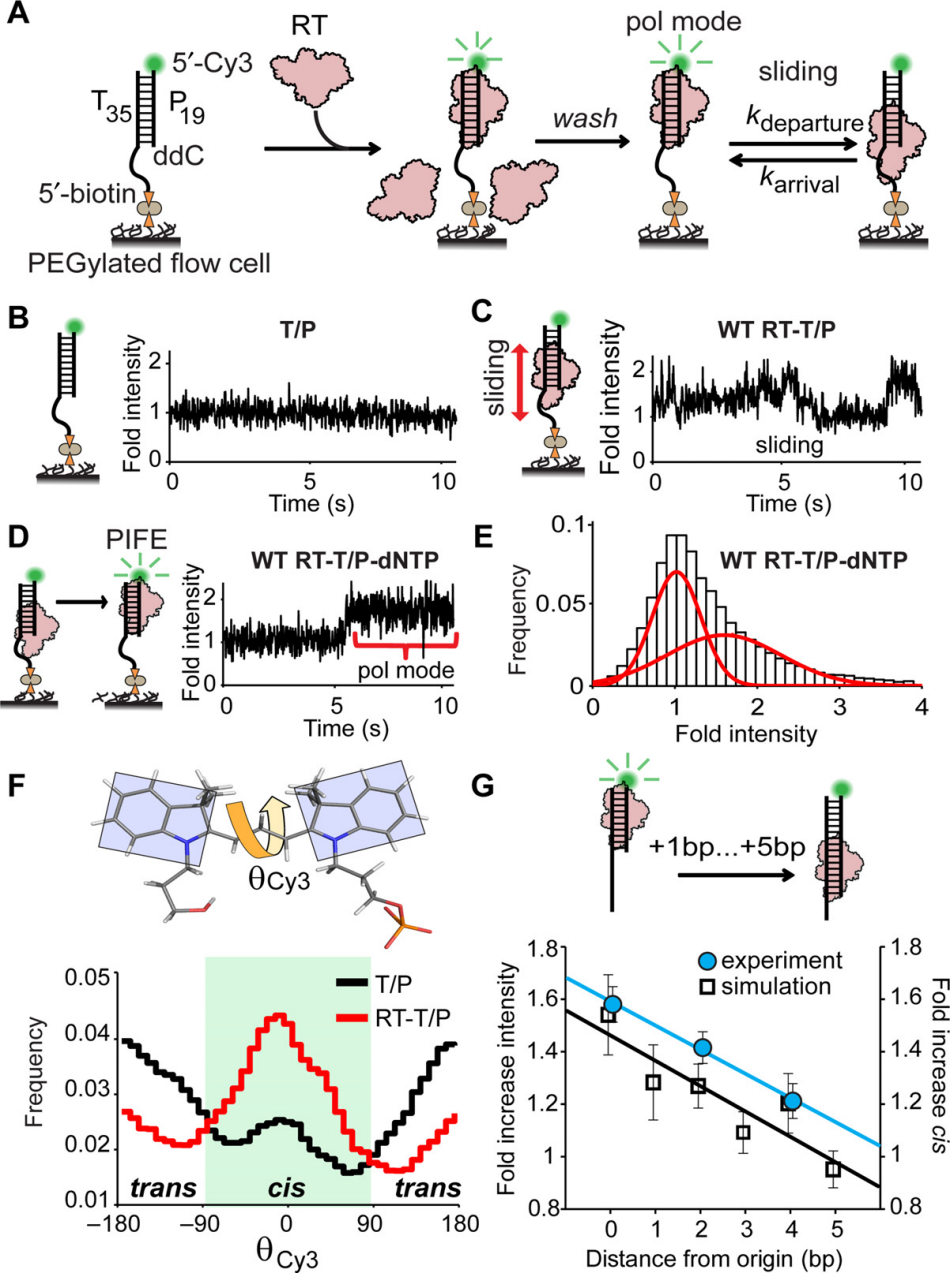
Single-molecule PIFE assay for RT sliding on the T/P. (**A**) Schematic of PIFE assay. (**B,C,D**) Representative PIFE traces for T/P, RT–T/P and RT–T/P–dNTP. (**E**) PIFE histogram from 106 different traces of the RT–T/P–dNTP complex showing two distinct populations. (**F**) θ_Cy3_ histograms from AMD simulations (*N*= 3 × 50 ns simulations per histogram). (**G**) Relationship between the PIFE signal (left ordinate), the *cis-*conformation of Cy3 (right ordinate) and the distance between RT and dye (abscissa). Data reported as mean ± SEM from at least three separate PIFE histograms (experiment) or AMD simulations (simulation).

We next quantitatively assessed the effect of K103N on the dynamic interactions between EFV–RT and the T/P substrate using single-molecule assays. To do so, we developed an assay to measure the PIFE (30–32) of Cy3 attached to the 5′ end of the primer (Figure 4A). PIFE intensity traces were detected with TIRF microscopy. In the absence of RT, there were extremely few observable fluctuations in the Cy3 intensity trajectories (Figure 4B). After the addition of RT, we observed fluctuating PIFE signals with transitions between 1- and 2-fold intensity (Figure 4C). Addition of dTTP to the RT–T/P complex resulted in stabilization of the PIFE signal into two discrete high and low states (Figure 4D and E). Since our fluorescence anisotropy experiments indicated that RT was sliding on T/P substrates (Figure 1) and since the apparent on/off rates were not consistent with association/dissociation events (Supplementary Figure S7), we considered these high and low states to likely result from sliding of RT on the T/P (Figure 4A).

To further understand the origin of our PIFE signal, we evaluated the mechanism by which RT enhances Cy3 fluorescence emission by performing AMD (33) simulations of the RT–T/P–dNTP ternary complex (20) in which we modeled Cy3 on the 5′ terminus of the primer. Cy3 was found to interact non-specifically with RT (Supplementary Figure S8A), markedly changed the distribution of θ_Cy3_, the dihedral angle between the planes of the heterocyclic rings of Cy3, enhancing the photoactive *cis*-isomer as predicted (34,35) (Figure 4F; Supplementary Figure S8B and C). When we extended the length of the DNA duplex in the structure of the RT–T/P–dNTP ternary complex and then performed AMD simulations, we observed an inverse relationship between the distance of RT from Cy3 and the fold-increase in *cis-*isomer (Figure 4G). Similarly, as we increased the length of the DNA/DNA duplex in the single-molecule PIFE assays, we observed a linear decrease in the intensity of the Cy3 signal (Figure 4G). These data indicate that the high PIFE signal is due to RT residing in a stable polymerase-competent RT–T/P–dNTP complex.

**Figure 5.**
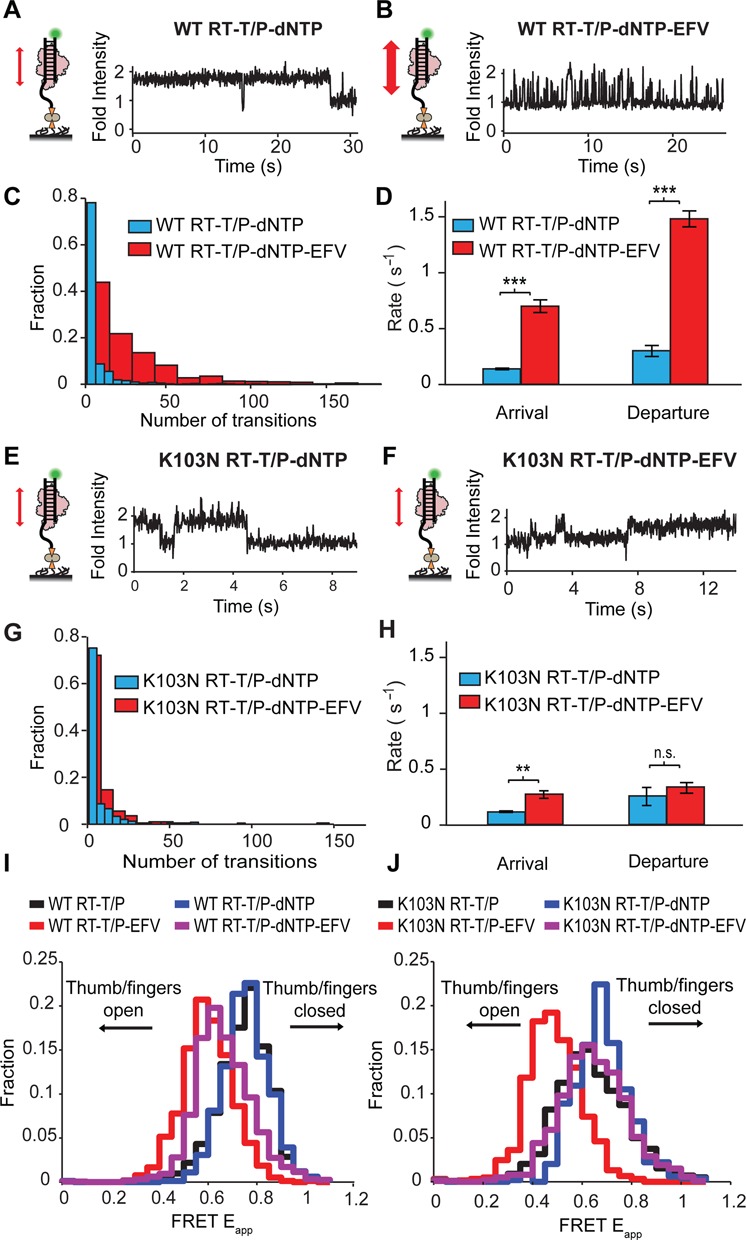
RT sliding and NNRTI-induced allostery are directed by thumb/fingers grip on the T/P substrate. (**A,B**) Representative PIFE traces for RT–T/P–dNTP and EFV-bound RT–T/P–dNTP. (**C**) PIFE transition histograms for the RT–T/P–dNTP complexes in the absence and presence of EFV. (**D**) Average *k*_arrival_ and *k*_departure_ rates extracted from 630 and 573 PIFE traces for the RT–T/P–dNTP complex in the absence and presence of EFV, respectively. Data are reported as mean ± SEM from at least three separate experiments. ***P < 0.001 (two-tailed *t*-test, equal variance). (**E,F**) PIFE traces for the K103N RT–T/P–dNTP and K103N–RT–T/P–dNTP–EFV complexes. (**G**) PIFE transition histogram for the K103N–RT–T/P–dNTP–EFV complex in the absence and presence of EFV. Data are reported as mean ± SEM from at least three separate experiments. (**H**) Average *k*_arrival_ and *k*_departure_ rates extracted from 926 and 725 PIFE traces for the K103N RT–T/P–dNTP complex in the absence and presence of EFV, respectively. Data reported as mean ± SEM. (**I**) FRET histograms for WT RT–T/P and RT–T/P–dNTP complexes in the absence and presence of EFV (*N* = 251, 147, 222 and 129 individual traces, respectively). (**J**) FRET histograms for K103NT RT–T/P and RT–T/P–dNTP complexes in the absence and presence of EFV (*N* = 178, 138, 156 and 150 individual traces, respectively).

We next assessed the affect of EFV on the stability of this complex. Upon infusion of EFV into the flow cell, we found that RT spent significantly less time in the polymerase-competent ternary complex (compare Figure 5A to B) and displayed many more transitions away from this state (Figure 5C). We calculated the rates of departure from (*k*_departure_) and arrival to (*k*_arrival_) the RT–T/P–dNTP complex (Supplementary Figure S9) and found that EFV significantly increased both (Figure 5D). We next assessed the interactions between K103N RT and the T/P substrate. The intensity traces for the K103N–RT–dNTP complex were found to be largely identical in the absence (Figure 5E) and presence (Figure 5F) of EFV. Furthermore, K103N RT displayed a similar number of transitions to and from the polymerase-competent ternary complex, both in the absence and presence of EFV (Figure 5G); consequently, *k*_departure_ and *k*_arrival_ were only minimally affected by the inhibitor (Figure 5H). Taken together, these data provide strong evidence that the EFV-bound K103N RT–T/P can form a stable ternary complex that facilitates phosphodiester bond formation.

### K103N promotes formation of an EFV-bound RT–T/P–dNTP ternary complex

We next introduced K103N into the p66 of the FRET RT construct and assessed the effect of EFV binding on conformational changes in the thumb/fingers. We found that addition of EFV to K103N RT–T/P also resulted in the opening of the fingers and thumb, with a change (Δ) in average *E*_app_ of 0.14 (Figure 5I), similar to the change observed in the WT complex (Δ*E*_app_ = 0.17; Figure 5J). However, the FRET histograms for K103N RT–T/P–dNTP were largely identical in the absence and presence of EFV (Figure 5J). Collectively, these results suggest that, rather than preventing inhibitor binding, K103N enables the inhibitor-bound enzyme to form a polymerase-competent ternary complex by maintaining its grip on the T/P substrate.

## DISCUSSION

**Figure 6.**
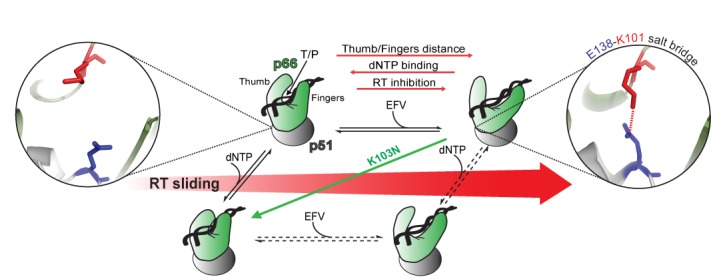
Model of EFV-induced allostery in HIV-1 RT. Cartoon models of the WT RT structures indicated with black text (PDBIDs correspond to those in Supplementary Table S1) and are placed on the horizontal axis as a function of the degree of sliding (red arrow). The p51 subunit and the p66 subunit are colored gray and green, respectively. The location of the E138-K101 salt bridge between the respective p51 and p66 domains is indicated by zoomed stick representation. The effect of the K103N mutation, which abrogates the E138-K101 salt bridge and stabilizes the polymerase competent form of RT, is signified by a green arrow.

Our data demonstrate a new allosteric mechanism by which EFV inhibits HIV-1 reverse transcription through the modulation of both intra-molecular conformational changes and the dynamic inter-molecular interactions of the RT–T/P and RT–T/P–dNTP complexes. We directly show that (i) EFV binding to RT induces opening of the fingers and thumb in the p66 subunit, which (ii) permits binding to the T/P but (iii) increases the dynamic sliding motion of the enzyme on its nucleic acid substrate and thus (iv) prevents dNTP binding. We further demonstrate (v) that each of these effects can be enhanced by the formation of a salt bridge between E138 (p51) and K101 (p66) and (vi) that the clinical NNRTI resistance mutation K103N restores the polymerase-competent intra- and inter-molecular RT dynamics, potentially by eliminating the salt bridge between K101 and E138. In our assays, the magnitude of the fingers–thumb opening, the increase in dynamic sliding and the inhibition of dNTP binding each corresponded with relative NNRTI susceptibility. We demonstrate that these observations are functionally interrelated, providing the first direct evidence that RT conformation directs its sliding dynamics on the T/P substrate, in turn, modulating polymerase activity. A model of our findings is presented in Figure 6.

Our intra-molecular spFRET studies demonstrate that the binding of EFV increases opening of the fingers and thumb in WT but not in K103N RT–T/P–dNTP complexes. Our anisotropy and PIFE data both show the consequence of the mutant's disruption of the EFV-induced conformational change: EFV-bound K103N retains its fingers/thumb grip on the T/P when dNTP is present and thus may stably reside in the functional polymerase-competent mode rather than slide unproductively along the T/P. Our spFRET results support the hypothesis that NNRTIs act as a ‘molecular wedge’ (27) in the critical hinge region between p51 and p66, with our data indicating that the E138-K101 salt bridge likely stabilizes these interactions to support an open RT conformation. Indeed, earlier intermolecular spFRET studies demonstrated that RT can slide and flip on nucleic acid substrates (12) and that T/P substrate composition directs the orientation of RT (13). These authors hypothesized that NNRTI binding and subsequent opening of the thumb/fingers domains of RT would result in sliding of RT away from the polymerase-competent configuration relative to an extending primer (12). Our results are consistent with this hypothesis and add new information about the dynamic interplay among the allosteric conformational changes induced by NNRTIs, dNTP binding and RT sliding.

Notably, the putative mechanism by which the K103N variant resists EFV inhibition (see (24) for a review; see also Supplementary Discussion) has thus far been based on experiments conducted in the absence of T/P or dNTP or both. We discovered a critical role for dNTP in the mechanism of EFV resistance in that inhibitor-bound K103N RT retained its grip on the T/P and reduced sliding only in the presence of dNTP. Furthermore, our findings challenge the current convention that drug resistance correlates with reduced drug binding affinity and shed light on a previously described anomaly: direct contacts between EFV and K103N are not observed, and a stereochemical basis for any effect on binding cannot be inferred from the crystal structures of WT or K103N RT in complex with EFV (25). Consistent with this, we observed no reduction of EFV binding affinity in the K103N mutant. In contrast, K103N did lower the binding affinity of NVP for the RT–T/P complex, demonstrating that a single mutation in RT can elicit different modes of resistance to structurally diverse NNRTIs. Of note, RPV retains activity against HIV-1 containing K103N (22). In the crystal structure of K103N RT in complex with RPV (25), the salt bridge between K101 and E138 remains intact, and our anisotropy assays show that RPV binds with increased affinity to the RT–T/P complex (Supplementary Figure S2). By showing that instead of targeting EFV binding affinity K103N may target the p51-p66 salt bridge in modulating the conformational dynamics of EFV-bound RT, in turn reducing enzyme sliding on the T/P substrate and permitting polymerization, our data highlight a potential critical role for this salt bridge in the global dynamics and subsequent function of RT.

We used complementary strategies to gain insight into the mechanisms of EFV inhibition and resistance. First, we developed a novel assay of RT mobility on nucleic acid substrates based on absolute fluorescence anisotropy that can be used to measure NNRTI and/or dNTP binding affinity—to our knowledge the first assay of its kind. This assay is easily implemented and should be readily adaptable to any protein–nucleic acid system of interest in which protein mobility may play a role. In contrast to other measures of protein–DNA binding such as gel-shift assays or activity-based techniques, we were able to simultaneously measure binding affinity and sliding dynamics with our assay, demonstrating that dNTPs and/or NNRTIs can alter RT mobility without an apparent change in binding affinity. We then simulated the interactions of RT bound to a T/P with a terminal Cy3 to characterize the RT sliding events observed in our PIFE assay, additionally providing insight into the molecular basis of PIFE.

Several key differences exist between our single-molecule experiments and those reported by other groups. Using a 19-bp DNA/RNA substrate, Liu *et al.* (12) did not observe a change in FRET signal between RT and the T/P substrate, whereas we did observe sliding on a dideoxy-terminated DNA/DNA duplex of identical length. This discrepancy highlights the distance sensitivity of our single-molecule PIFE assay compared with intermolecular spFRET: in their experiments, if RT were to slide up to 10 bp away from the duplex center (as predicted in our anisotropy model), a deviation from a high FRET signal would likely not be observed, since this distance (∼2.4 nm for A-form DNA/RNA) lies outside of the quasi-linear response regime for FRET between Cy3 and Cy5. We confirmed the PIFE signal resulting from the polymerase-competent RT configuration using AMD simulations of our system. The primary disadvantage of using PIFE to measure sliding is that we cannot observe distance distributions beyond the regime reported here (∼0–5 bp). We also cannot measure the possible effects of NNRTIs on RT flipping. Our PIFE assay detects the proximity of RT to the Cy3 dye without providing orientation information, as in Liu *et al.* (12); RT remains associated with the T/P during a flipping event, which would maintain the RT-Cy3 interaction and high PIFE signal. Moreover, in a previous study by Marko *et al.* (36), PIFE signals from RT–T/P complexes were interpreted as RT association/dissociation events, but these authors did not present experimental evidence indicating that they could distinguish between sliding and association/dissociation.

In our anisotropy assay, we assessed dNTP binding to an RT–T/P complex that cannot undergo catalysis because the DNA primer is chain-terminated, such that we captured both productive and non-productive dNTP binding. Consistent with our data, recent isothermal titration calorimetric (ITC) analyses revealed that the NVP-bound RT–T/P complex could not bind the incoming dNTP (26); in contrast, pre-steady-state kinetics data suggest that NNRTIs do not impede dNTP binding but instead inhibit DNA polymerization (5,6). However, thermodynamic (anisotropy and ITC) analyses and pre-steady-state kinetic assays measure different binding events; the pre-steady-state *K*_d_ predominantly reflects productive dNTP binding to an RT–T/P complex actively undergoing catalysis, capturing only rare kinetic events from RT–T/P complexes that are not NNRTI bound.

In summary, our data demonstrate that the dynamics of RT on its T/P substrate and subsequent activity are directly controlled by conformational dynamics between the thumb and fingers subdomains. Further, the data indicate that the EFV-mediated formation of a salt bridge between the RT side chains of E138 (p51) and K101 (p66) in a p51/p66 hinge region may help stabilize the thumb and fingers subdomains of RT in an open state and significantly contributes to the inhibitory capability of EFV in wild-type HIV-1, suggesting that the exploitation of this functionally important conformation should be pursued in future drug development. Future investigations are required to substantiate the extent of the role of the E138-K101 salt bridge in NNRTI mechanism. Our findings hint at the complex nature of allosteric inhibition and provide new insight into the interrelatedness of residue-scale interactions, global conformational change, DNA–protein dynamics and enzymatic activity.

## SUPPLEMENTARY DATA

Supplementary Data are available at NAR Online.

SUPPLEMENTARY DATA
