# Supplementary material for: Mechanism of allosteric inhibition of HIV-1 reverse transcriptase revealed by single-molecule and ensemble fluorescence
Source: Nucleic Acids Res. 2014 Sep 17;42(18):11687–96. doi: 10.1093/nar/gku819 (PMC4191400; doi:10.1093/nar/gku819)
Supplement: SUPPLEMENTARY DATA [file supp_42_18_11687__index.html]

Mechanism of allosteric inhibition of HIV-1 reverse transcriptase revealed by single-molecule and ensemble fluorescence — Mechanism of allosteric inhibition of HIV-1 reverse transcriptase revealed by single-molecule and ensemble fluorescence — SUPPLEMENTARY DATA 

# Mechanism of allosteric inhibition of HIV-1 reverse transcriptase revealed by single-molecule and ensemble fluorescence

## SUPPLEMENTARY DATA

**Files in this Data Supplement:**

- SUPPLEMENTARY DATA
- SUPPLEMENTARY DATA
