# Supplementary material for: Mechanism of allosteric inhibition of HIV-1 reverse transcriptase revealed by single-molecule and ensemble fluorescence
Source: Nucleic Acids Res. 2014 Sep 17;42(18):11687–96. doi: 10.1093/nar/gku819 (PMC4191400; doi:10.1093/nar/gku819)
Supplement: SUPPLEMENTARY DATA [file supp_gku819_nar-01247-f-2014-File008.pdf]

Supplementary Information

Supplementary Figures

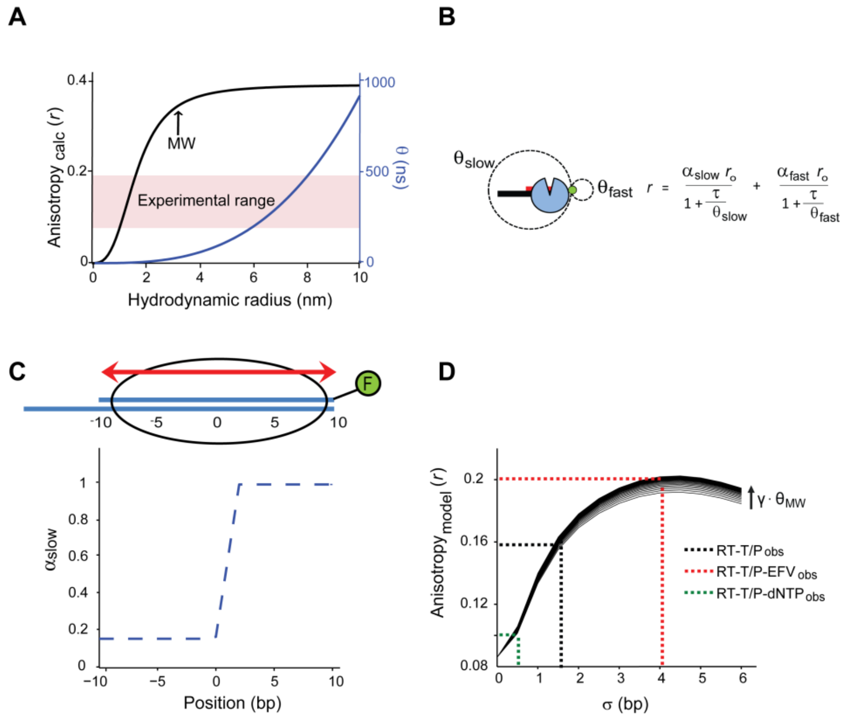

Supplementary Figure 1: Determination of the distribution of RT on the T/P substrate from anisotropy (*r*) data. A) Predicted response of *r* and θ to hydrodynamic radius. The hypothetical *r* value for the RT-T/P complex based on molecular weight (MW) is indicated by arrow. The range for the experimentally observed *r* values from Figure 1a is highlighted in pink. **B)** Schematic illustrating the slow (θ_{slow}) and fast (θ_{fast}) tumbling of the fluorescence dye attached to the T/P substrate. The Perrin equation which includes contributions from θ_{slow} and θ_{fast} is also shown. **C)** A simplified model of RT sliding (black oval) on the T/P substrate (blue lines). If RT moves toward the +10 position, it interacts with the fluorescein dye (green). A plot of α_{slow} as a function of the position of RT on the T/P substrate is also shown. **D)** Relationship between *r* values, predicted from the model in (**C**), and the standard deviations (σ) for the distributions of RT on the T/P. The response to changes in θ is dependent on the constant γ, where $1 < \gamma < 10$. Values of σ derived were derived for the experimentally determined r values for the RT-T/P, RT-T/P-dNTP and RT-T/P-EFV complexes (dotted lines) are were used to plot Figure 1b).

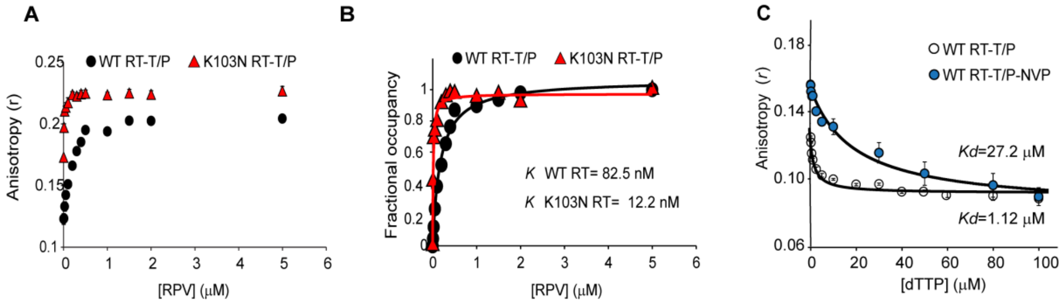

Supplementary Figure 2: Supplementary anisotropy data. RPV binding to WT and K103N RT is shown as **(A)** absolute anisotropy or **(B)** normalized binding curves. **(C)** dTTP binding to WT RT-T/P complexes in the absence and presence of NVP measured by anisotropy. Data reported as mean ± SEM.

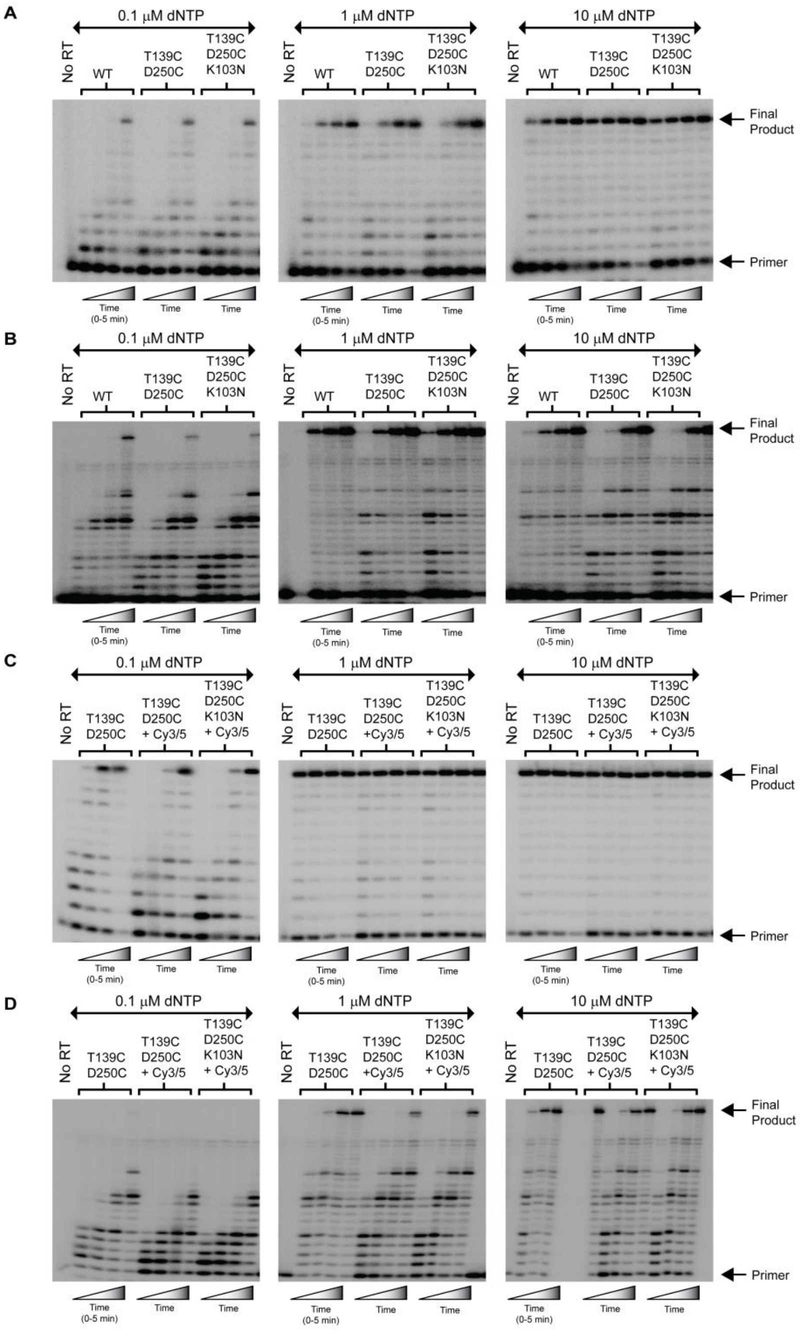

Supplementary Figure 3: Neither the mutations nor chemical modifications of the FRET RT constructs significantly impairs the DNA- and RNA-dependent DNA polymerase activities of the enzymes. A) Representative autoradiograph of the DNA-dependent DNA polymerase activity of WT and $p66^{C38V/T139C/D250C/C280S}/p51^{C38V/C280S}$ (T139C/D250C) RT in the presence of 0.1, 1 and 10 μM dNTP. Reaction times ranged from 0 – 5 min. **B)** Representative autoradiograph of the RNA-dependent DNA polymerase activity of WT and $p66^{C38V/T139C/D250C/C280S}/p51^{C38V/C280S}$ (T139C/D250C) RT in the presence of 0.1, 1 and 10 μM dNTP. Reaction times ranged from 0 – 5 min. **C)** Representative autoradiograph of the DNA-dependent DNA polymerase activity of unlabeled or Cy3/Cy5-dual labeled $p66^{C38V/T139C/D250C/C280S}/p51^{C38V/C280S}$ (T139C/D250C) RT in the presence of 0.1, 1 and 10 μM dNTP. Reaction times ranged from 0 – 5 min. **D)** Representative autoradiograph of the RNA-dependent DNA polymerase activity of unlabeled or Cy3/Cy5-dual labeled $p66^{C38V/T139C/D250C/C280S}/p51^{C38V/C280S}$ (T139C/D250C) RT in the presence of 0.1, 1 and 10 μM dNTP. Reaction times ranged from 0 – 5 min.

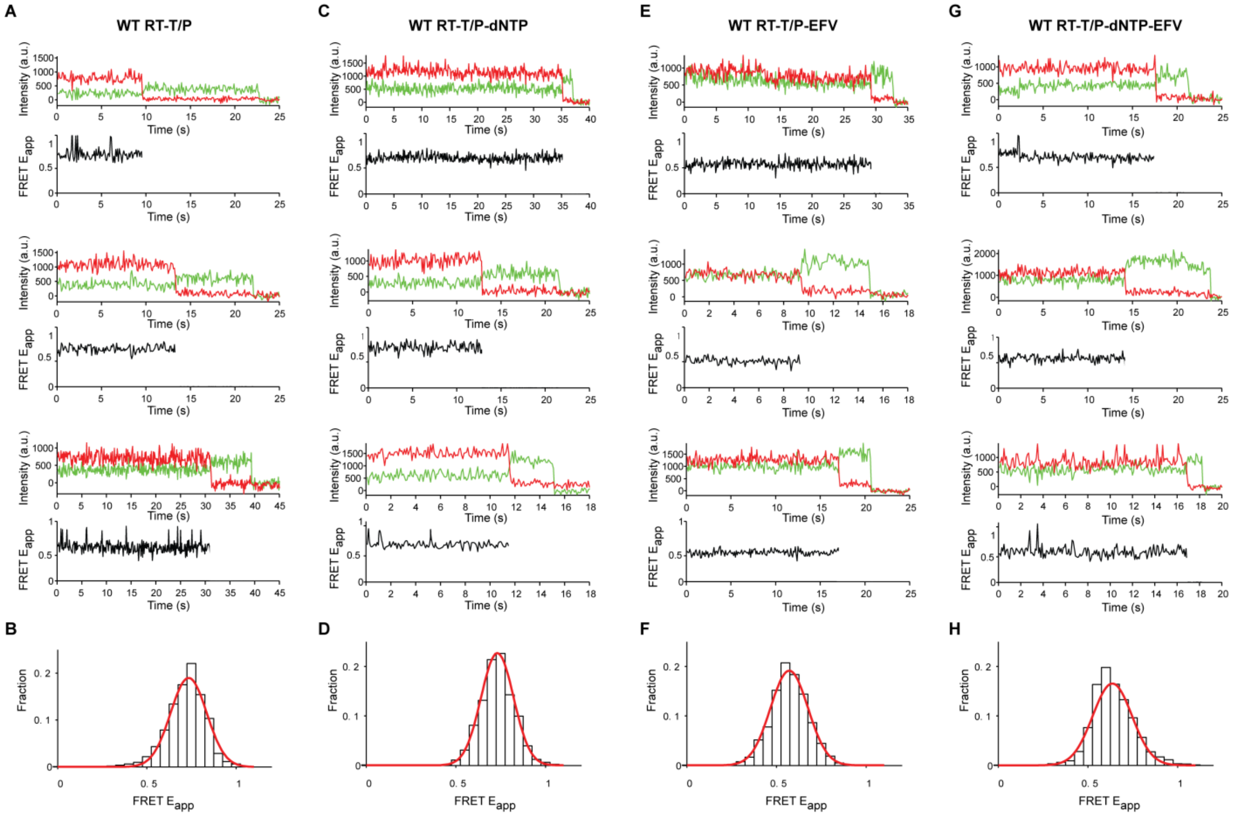

Supplementary Figure 4: Representative FRET data for the RT-T/P, RT-T/P-dNTP, RT-T/P-EFV and RT-T/P-dNTP-EFV complexes. A) 3 representative FRET traces are shown for Cy3- and Cy5-labeled RT in complex with T/P. The donor and acceptor intensity trajectories are shown in green and red, respectively. The E_{app} calculated from each of the traces is shown in black. The E_{app} traces were truncated at the moment of acceptor photobleaching. For clarity, we primarily included long-lived FRET signals. **B)** FRET histogram for the RT-T/P complex (N = 251). **C)** 3 representative FRET traces are shown for Cy3- and Cy5-labeled RT in complex with T/P and dNTP. The donor and acceptor intensity trajectories are shown in green and red, respectively. The E_{app} calculated from each of the traces is shown in black. **D)** FRET histogram for the RT-T/P-dNTP complex (N = 147). **E)** 3 representative FRET traces are shown for Cy3- and Cy5-labeled RT in complex with T/P and EFV. The donor and acceptor intensity trajectories are shown in green and red, respectively. The E_{app} calculated from each of the traces is shown in black. **F)** FRET histogram for the RT-T/P-EFV complex (N = 229). **G)** 3 representative FRET traces are shown for Cy3- and Cy5-labeled RT in complex with T/P, dNTP and EFV. The donor and acceptor intensity trajectories are shown in green and red, respectively. The E_{app} calculated from each of the traces is shown in black. **H)** FRET histogram for the RT-T/P-dNTP-EFV complex (N = 129). For (**B**, **D**, **G**, **H**) the red lines indicate single Gaussian fits to the data.

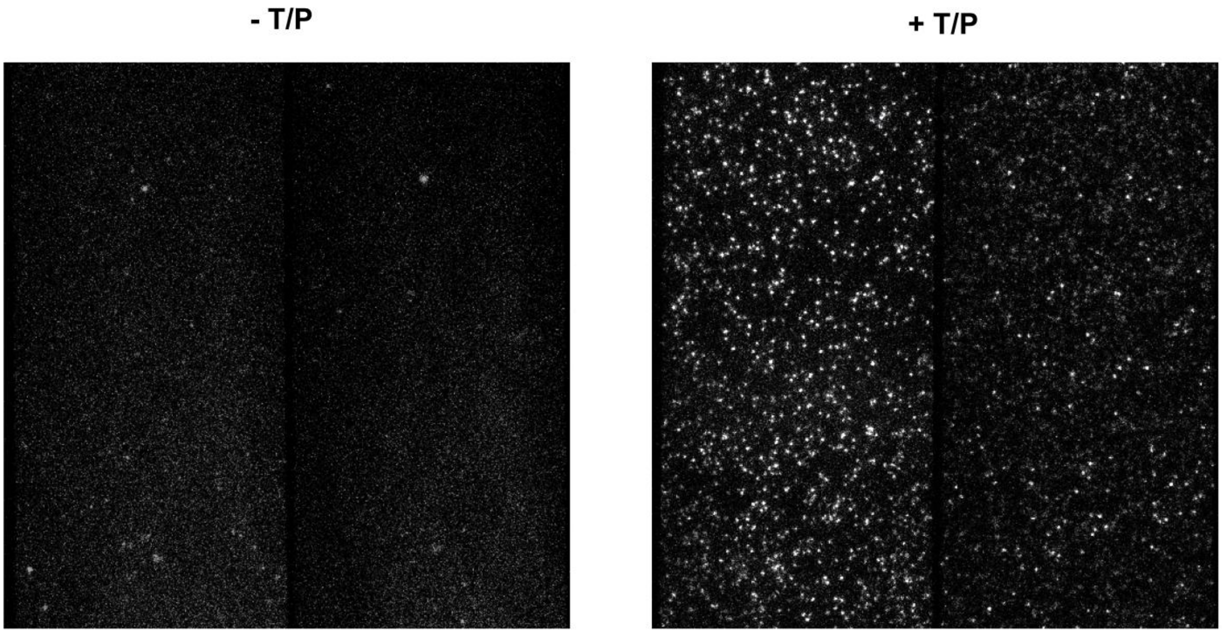

Supplementary Figure 5: RT-Cy3/5 constructs bind specifically to T/P substrates. The field of view from the TIRF microscope (left channel: donor, right channel, acceptor) is shown for 40 pm of WT RT-Cy3/5 injected into **Left)** blank flow cells containing PEG, biotin-PEG, and neutravidin or **Right)** T/P-tethered flow cells containing PEG, biotin-PEG, neutravidin, and biotin-T/P.

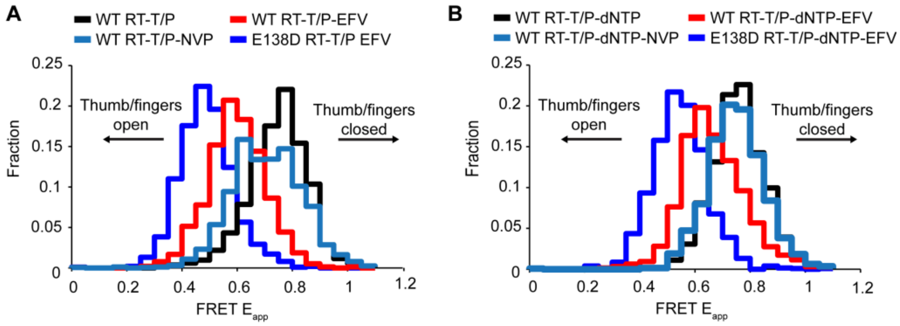

Supplementary Figure 6: Supplementary FRET data. (B) FRET histograms for WT RT-T/P in the absence (N = 251 individual traces) or presence of NVP (N = 154) or EFV (N = 147). A FRET histogram for the E138D RT-T/P complex in the presence of EFV is also shown (N = 107). **(C)** FRET histograms for WT RT-T/P-dNTP in the absence (N = 222 traces) or presence of NVP (N =144) or EFV (N = 129). A FRET histogram for the E138D RT-T/P-dNTP complex in the presence of EFV is also shown (N = 94).

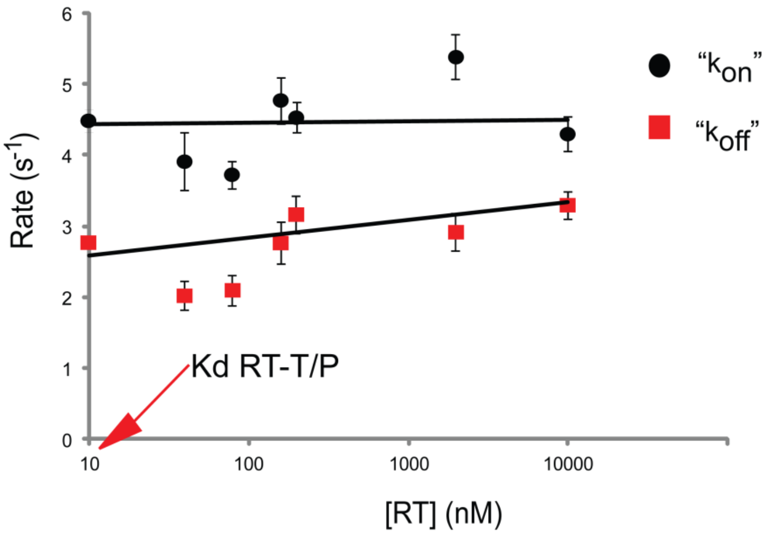

Supplementary Figure 7: PIFE fluctuations are not due to RT-T/P association/dissociation events. PIFE fold-intensity is shown as a function of RT concentration. Each data point represents the mean ± SEM taken from at least three exponential fits of an average of 229 ± 104 (mean ± SD) traces. If association/dissociation was dominating the observed PIFE rates, an apparent k_{off} that was independent of RT concentration and a k_{on} that crossed k_{off} at the K_d of the RT-T/P complex (~9 nM; Figure 1) would be expected. However even at RT concentrations higher than K_d x 10^3, the apparent k_{on} was not greater than the apparent k_{off}.

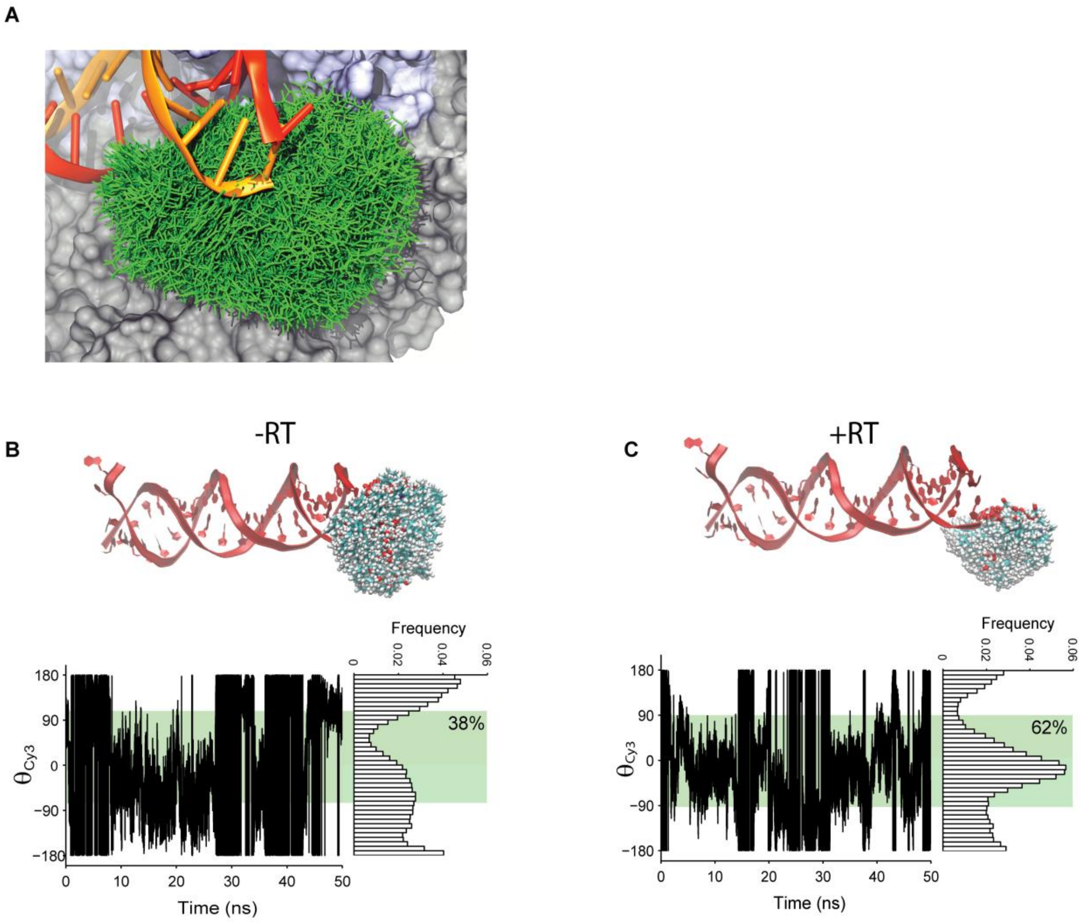

Supplementary Figure 8: The *cis* isomer of Cy3 is stabilized through interaction with RT. A) Snapshots (100 ps increments) of the structure of Cy3 (green sticks) from a single 50 ns AMD simulation of the RT-T/P-dNTP ternary complex. The p66 and p51 subunits of RT are colored light and dark grey, respectively. The template and primer strands are colored red and orange, respectively. **B,C)** Representative θ_{Cy3} trajectories of the 5'-Cy3 labeled T/P are show in the absence **(B)** and presence **(C)** of RT. In the θ_{Cy3} histograms, the *cis* isomer region is highlighted in green.

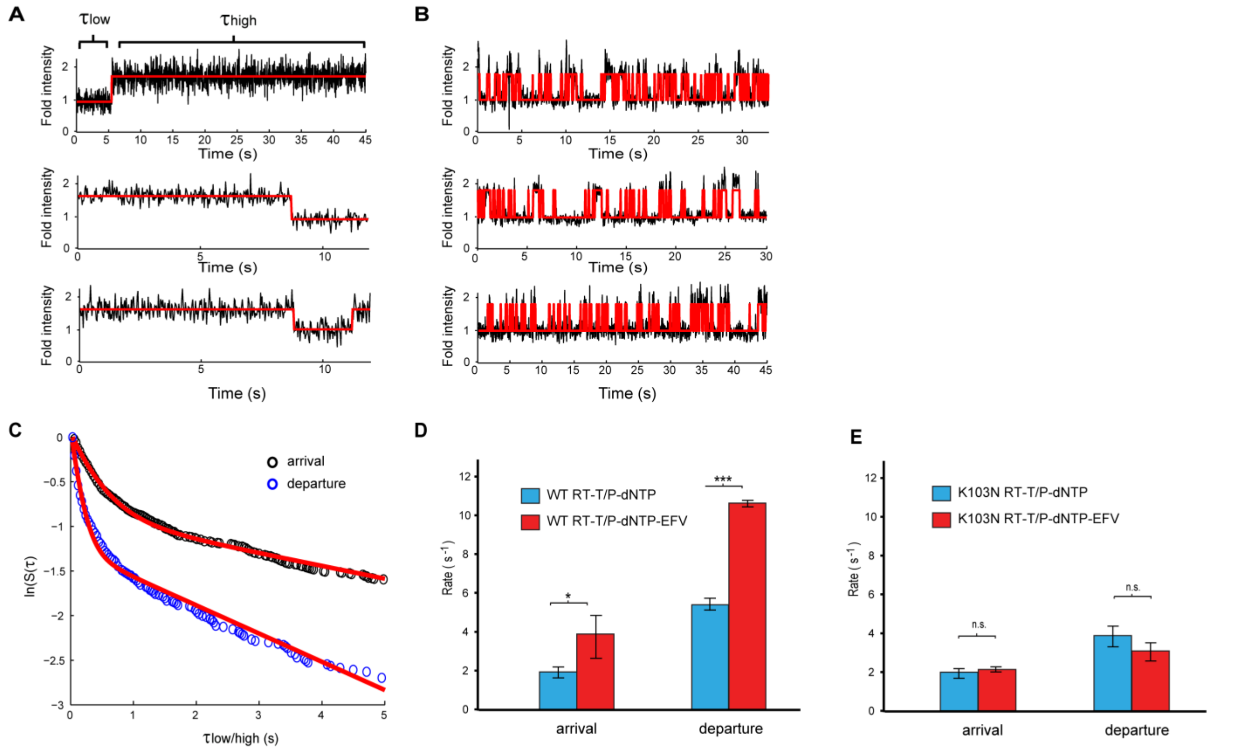

Supplementary Figure 9: Rates or arrival and departure from the RT-T/P-dNTP complex in the absence and presence of EFV. A) Representative single-molecule PIFE traces for the WT RT-T/P-dNTP complex. The red lines indicate fitted idealized states (see Methods). Definition of high (τ_{high}) and low (τ_{low}) dwell times are shown. **B)** Representative single-molecule PIFE traces for the WT RT-T/P-dNTP complex in the presence of EFV. The red lines indicate fitted idealized states (see Methods). **C)** Representative slow and fast rates of arrival and departure ($k_{arrival}$ and $k_{departure}$) for the WT RT-T/P-dNTP complex. The plot of the log of the survival function for the WT RT-T/P-dNTP complex, ln(Sτ), versus τ values for arrival or departure is biphasic and is best-fit to a double-exponential curve ($R^2 \geq 0.99$), whereas a linear dependence between these values would have indicated only one dominant rate. Because the slow rates dominated the S(τ) distributions (~90% of total before EFV and ~75% of total after EFV) and were most affected by EFV, we reported these in Figure 6d. **D)** The fast rates of $k_{arrival}$ and $k_{departure}$ from the WT RT-T/P-dNTP complex in the absence (N = 630 traces) and presence (N= 573 traces) of EFV. **e**, The fast rates of $k_{arrival}$ and $k_{departure}$ from the K103N RT-T/P-dNTP complex in the absence (N = 926 traces) and presence (N = 725 traces) of EFV. For **(D)** and **(E)**, * P<0.05, *** P<0.001, n.s., not significant (two tailed t-test, equal variance).

Supplementary Movie 1: 3D visualization of EFV-bound HIV-1 RT and the NNRTI binding pocket. WT RT in complex with EFV (1FK9) is shown as a solvent-accessible surface; p51 is shown in brown, p66 in green. The movie begins with a view of the empty T/P binding groove flanked by the thumb (left) and fingers (right), and subsequently zooms into the NNRTI binding pocket containing EFV (yellow). K103N, K101, and E138 are indicated. Next, an overlay of EFV-bound K103N RT (1FKO) appears in lighter colors, highlighting the disruption of the E138-K101 salt bridge by K103N. Lastly, the camera zooms out to show the salt bridge in the context of the thumb and fingers subdomains.

Supplementary Materials and Methods

RT constructs for PIFE and anisotropy assays

K101R, K103N and E138D were introduced into the WT p6HRT-Prot prokaryotic expression vector (1) by site-directed mutagenesis using the QuikChange mutagenesis kit (Stratagene). WT, K101R, K103N, E138D and E138D/K101R HIV-1 RTs were purified as described previously (2, 3). Protein concentration was determined spectrophotometrically at 280 nm using an extinction coefficient (ε_{280}) of 260450 M^{-1} cm^{-1}.

RT constructs for single-pair FRET

C38V and C280S were introduced into the WT p6HRT-Prot prokaryotic expression vector as described above. The gene from the RT p51 gene was then amplified and cloned as an N-terminal hexahistidine fusion protein into MSC2 of the pETDuet-1 vector (Novagen). We also generated a C38V/E138D/C280S construct that was cloned into MCS2 of pETDuet-1. T139C and D250C were introduced into the backbone of the C38V/C280S p6HRT-Prot prokaryotic vector, and the RT p66 gene was amplified and cloned into MCS1 of pETDuet-1. The C38V/K103N/T139C/D250C/C280S RT p66 construct was also cloned into MCS1 of pETDuet-1. The $p66^{C38V/T139C/D250C/C280S}/p51^{C38V/C280S}$, $p66^{C38V/K103N/T139C/D250C/C280S}/p51^{C38V/C280S}$ and $p66^{C38V/T139C/D250C/C280S}/p51^{C38V/E138D/C280S}$ RTs were purified as described previously (4). RT was labeled for 1 hr at 37 ° C in 50 mM Tris-HCl, pH 8.2, 25 mM NaCl, 1mM EDTA and 1 mM TCEP-HCl by addition of a 10-fold molar excess of a solution containing equimolar concentrations of Cy3- and Cy5-maleimides (GE Healthcare Life Sciences). The labeled protein was separated from the excess dyes by gel filtration on an Illustra NAP-5 column (GE Healthcare Life Sciences). The efficiency of cysteine labeling, as determined by UV spectroscopy using extinction coefficients of 150,000 M^{-1} cm^{-1} for Cy3 and 250, 000 M^{-1} for Cy5, ranged from 70-80%.

T/P substrates

All synthetic oligonucleotides were purchased from Integrated DNA Technologies. For the single-molecule PIFE experiments, a 5'-Cy3 labeled 19 nucleotide DNA primer (5'-Cy3-

CAGTCCCTGTTCGGGCGC-ddC-3’) was annealed to a 5’-biotinylated 35 nucleotide DNA template (5’-biotin-GGGTTTGCTAAGCACCGGCGCCCGAACAGGGACTG-3’). We also performed PIFE experiments using a 21 nucleotide DNA primer (5’-Cy3-CAGTCCCTGTTCGGGCGCCGCT-ddC-3’) or a 23 nucleotide DNA primer (5’-Cy3-CAGTCCCTGTTCGGGCGCCGCTCT-ddC-3’) annealed to the same template. For the single-pair FRET experiments, the same T/P was used, but it lacked 5’-Cy3. For the anisotropy experiments we used a 19 nt DNA primer (5’-Fluorescein-dT-CAGTCCCTGTTCGGGCGC-ddC-3’) that was annealed to the 35 nucleotide DNA template that lacked a biotin label.

Fluorescence anisotropy

Fluorescence anisotropy experiments were performed as previously described (5, 6), using a Varian Cary Eclipse fluorescence spectrophotometer. The excitation and emission wavelength were set at 485 and 520 nm, respectively, and the excitation and emission slit widths were set at 5 and 10 nm respectively. The concentration of T/P in all experiments was 5 nM (in a 400 μL cuvette). Anisotropy (r) was calculated as:

$$r = (I_{VV} - G \cdot I_{VH})/(I_{VV} + 2 \cdot G \cdot I_{VH}) \quad (1)$$

where I_{VV} is the fluorescence intensity with vertically oriented excitation and emission polarizers and I_{VH} is the fluorescence intensity with a vertically oriented excitation polarizer and a horizontally oriented emission polarizer. The G-factor, defined as $G=I_{HV}/I_{HH}$, was measured before each experiment to ensure a value of ~ 1.65. Anisotropy values were collected in triplicate using an integration time of 2.0 s. K_d values were calculated by fitting curves to a standard single-site binding equation using Origin (OriginLab).

Anisotropy calculations

For all theoretical calculations, r was calculated using the Perrin equation:

$$r = r_o/(1+\tau/\theta) \quad (2)$$

where r_o is the fundamental anisotropy, τ the lifetime and θ the rotational correlation time (7). For fluorescein, empirically determined values of $r_o = 0.39$ ns and $\tau = 4.1$ ns were used (8, 9). θ was calculated using the equation:

$$\theta = \eta V/RT \quad (3)$$

where η is the viscosity of water in poise (0.890 cP for all calculations), V is the volume of the molecular complex, R the gas constant and T temperature (298.15 K for all calculations). For calculations of hydrodynamic radius, the radius was derived directly from the volume term assuming a spherical RT-T/P complex. To determine θ by molecular weight (MW), V was further defined as :

$$V=MW(\nu+h) \quad (4)$$

where MW is the molecular weight of the RT-T/P complex (130 kD), ν the specific volume (0.73 ml/g for all calculations), and h is the hydration (0.23 g H_2O/g protein) (7). For calculations involving the segmental motion of the fluorophore, the Perrin equation was further broken down into

$$r=\alpha_{slow} \cdot r_o/(1+\tau_{slow}/\theta_{slow}) + \alpha_{fast} \cdot r_o/(1+\tau_{fast}/\theta_{fast}) \quad (5)$$

which is a linear combination of the anisotropy resulting from the slow rotational correlation time from the complex (θ_{slow}) and the fast rotation of the fluorescent dye itself (θ_{fast}). α_{slow} and α_{fast} are respective scaling factors for θ_{slow} and θ_{slow}, where $\alpha_{slow} + \alpha_{fast} =1$.

Single-molecule TIRF microscopy

Single-molecule TIRF microscopy was performed as described previously (10). We used an inverted fluorescence microscope (Olympus IX71) that was modified for prism-based TIRF and coupled to a 532 nm diode laser (CrystaLaser) and a 647 nm diode laser (Melles Griot). Fused silica slides (G. Finkenbeiner, Inc.) were surface-passivated with PEG and biotin-PEG (Laysan Bio., Inc.), as described previously (11). Fluorescence from individual tethered complexes was sent through a 550 longpass filter and, using a DualView apparatus (Photometrics), was split into Cy3 or Cy5 emission pathways by a 610 nm dichroic mirror, respectively filtered by either a 580/40 nm bandpass filter or a 660 nm longpass filter (Chroma Technology Corp.), and subsequently imaged onto two halves of an electron-multiplying CCD (EMCCD) camera (Andor Technologies). Signals were identified by intensity thresholding and by goodness of fit of 7 × 7 pixel peaks to a 2D Gaussian. Time-dependent intensity traces were extracted and corrected for local background. All analyses of single-molecule data were performed using custom-written software in MATLAB (The Mathworks, Inc.).

Buffer composition

All experiments were performed in 50 mM Tris-HCl, pH 7.5, 25 mM NaCl, and 5 mM $MgCl_2$. The buffer used in TIRF microscopy experiments also contained 0.1 mg/ml glucose oxidase, 0.02 mg/ml catalase, 0.4% wt/v β-d-glucose, and 2 mM Trolox. Unless otherwise indicated, the TTP, EFV and NVP concentrations were held at 50 μM, 500 nM and 1 μM, respectively, ensuring saturating binding conditions for all ligands.

Single pair FRET

Unlabeled T/P was surface tethered by introducing 2 μM T/P into the flow cell to allow formation of a surface-dense layer of T/P substrates, which promoted observation of RT-T/P complexes. 40 pM of labeled RT was then introduced into the flow cell and allowed to bind to the T/P. Unbound RT was removed by extensive washing. Low concentrations of RT followed by a wash step eliminated excess background noise and/or saturation of the EMCCD. Based on the relative distribution of donor and acceptor intensities observed in the TIRFM fields of view, the dual-labeling efficiency of HIV-1 RT was ~ 10 % for all constructs assessed. Cy3 was excited with ~1.5 mW 532 nm light, and data was acquired at 10 Hz. Single-molecule peak choice was thresholded on Cy5 intensity such that only molecules exhibiting FRET were analyzed. E_{app} was calculated using the equation:

$$E_{app} = I_A/(I_D+I_A) \quad (6)$$

where I_D and I_A denote background-corrected donor and acceptor intensities, respectively. Note that E_{app} should be independent of the relative arrangement of Cy3 or Cy5 on the dual-labeled RT. E_{app} histograms were subsequently constructed by collecting the pre-photobleach regions of all traces visibly resulting in donor and acceptor photobleaching, binned into 25 bins, and then fit with single Gaussian functions (see Supplementary Figure 3). Thumb/fingers distances reported in Supplementary Table 1 were derived from the fitted E_{app} values using the Förster equation:

$$E_{app}=1/(1+(r/R_o)^6) \quad (7)$$

where r is the distance between the dipoles of Cy3 and Cy5, and R_o is the empirically calculated Förster distance for the Cy3-Cy5 dye pair (54 Å; adapted from Anderson et al., 2008).

Single-molecule PIFE

All PIFE data was acquired on a 512 × 256 pixel region of the EMCCD at 33 Hz. T/P molecules were surface tethered to the PEGylated flow cell via a biotin:streptavidin:biotin-PEG linkage. The concentrations of T/P was typically 20 pM, which provided an optimal surface density of ~100 molecules per field of view. 250 nM RT was introduced into the flow cell and incubated for 5 min, followed by a wash step with 5X volume of imaging buffer. The power of the 532 nm excitation beam was ~6 mW. Fluorescence intensity traces which yielded visible fluctuations and exhibited single-step photobleaching were selected for analysis. These were rescaled to "fold intensity" PIFE traces by fitting each trajectory to a two-component Gaussian function and by dividing the raw intensity data by the fitted mean of the lowest Gaussian peak. PIFE histograms were constructed by binning PIFE traces into 50 bins and fitting them with a two-component Gaussian function to extract the average fold intensity increase. Data were also rescaled for use with the vbFRET program (12), and fluctuating fluorescence intensity traces were fit to idealized states. Idealized dwell times (τ) were aggregated into survival probability distributions $S(\tau)$ to eliminate any model bias from choice of bin number. Distributions were fit with double exponential functions to calculate sliding rates.

AMD simulations of PIFE

MD simulations were performed in NAMD (13) with the atomic coordinates of the ternary RT-T/P-TTP complex (PDB: 1RTD) modified at the 5'-end of the primer with the coordinates of the first excited state of Cy3 optimized at the CIS/6-31G(d,p) level (14) (coordinates courtesy of David Norman; University of Dundee). Systems were solvated in a 125 Å x 96 Å x 96 Å periodic box of TIP3 waters, ionized with 25 mM NaCl, and loaded with the CHARMM27 force field, including Cy3 parameters courtesy of Dr. van der Vaart (University of South Florida). As previously described (15), Spiriti *et al*. altered the dihedral parameters of the polymethine chain connecting the heterocyclic rings to be less flexible in order to explicitly prevent excessive Cy3 isomerization. Upon request, Dr. van der Vaart generously provided the original, flexible dihedral parameters for the linker. For simulations in which the distance between the Cy3 and RT were increased, the DNA duplex was rotated 35.3° and translated 3.4 Å upstream along its principle axis for each 1 base pair addition. Periodic boxes were resized as necessary (total size ranging from ~100,000-125,000 atoms). Systems were subjected to 100,000 steps of conjugate

gradient minimization, heated to 300 K in increments of 30 K over the course of 1 ns, and allowed to equilibrate for an additional 1 ns in an NPT ensemble with traditional MD. For every system simulated, the NAMD parameters for AMD implementation, accelMDE and accelMDalpha were defined as accelMDE=V_d +4·N_{res} and accelMDalpha=4·(N_{res}/5), where V_d is the average of the dihedral potential over the course of the 1 ns of MD equilibration, and N_{res} is the number of residues in the system. For the MD portion, 0.05 kcal/mol·$Å^2$ harmonic constraints were added to all protein Cα and DNA P atoms. For the AMD portion, all constraints within 25 Å of any Cy3 atom were subsequently removed. All simulations were run with 2 fs time steps (employing the SHAKE algorithm), Particle-mesh Ewald electrostatics, a Langevin Thermostat set to 300 K and a Langevin Barostat set to 1.01325 atm. θ_{Cy3} was defined as the dihedral angle between the Cy3 atoms C5A, C5B, N1A and N1B. θ_{Cy3} trajectories were analyzed with VMD (16). For each system simulated, 50 ns AMD simulations were performed in triplicate, for a total simulation time of 0.9 μs.

Supplementary Table 1: E_{app} and distance values determined from single-pair FRET experiments

Complex	E_{app}[a]	Averaged Cy3-Cy5 FRET-derived distances (Å)[b,c]	D250-T139 Cα-Cα crystal structure distance (Å) (PDBID)
WT RT-T/P	0.74 ± 0.14	45.6 ± 3.1	51.6 (2HMI)
WT RT-T/P-dNTP	0.73 ± 0.13	45.7 ± 2.7	50.6 (1RTD)
WT RT-T/P-EFV	0.57 ± 0.15	51.5 ± 1.7	59.6 (43BO)[d]
WT RT-T/P-dNTP-EFV	0.64 ± 0.15	49.2 ± 2.3	-
WT RT-T/P-NVP	0.68 ± 0.18	47.6 ± 3.2	56.2 (3V81)
WT RT-T/P-dNTP-NVP	0.72 ± 0.14	46.1 ± 2.9	
K103N RT-T/P	0.60 ± 0.19	50.4 ± 2.4	-
K103N RT-T/P-dNTP	0.65 ± 0.14	48.8 ± 2.1	-
K103N RT-T/P-EFV	0.46 ± 0.15	55.6 ± 1.2	-
K103N RT-T/P-dNTP-EFV	0.62 ± 0.18	49.8 ± 2.5	-
E138D RT-T/P	0.69 ± 0.14	47.2 ± 2.6	-
E138D RT-T/P-dNTP	0.66 ± 0.19	48.2 ± 3.1	-
E138D RT-T/P-EFV	0.46 ± 0.12	55.6 ± 1.0	-
E138D RT-T/P-dNTP-EFV	0.52 ± 0.13	53.3 ± 1.3	-

[a] Reported as the mean ± SD from single Gaussian fits (all fits $R^2 \geq 0.99$).

[b] Approximate values derived from E_{app} (see Methods). Reported as mean ± SD.

[c] $P < 0.02$ between all mean value pairs

[d] RNA/DNA T/P substrate

Model for the distribution of RT on T/P substrate based on anisotropy data

To account for the observed changes in *r* for the RT-T/P complex upon binding TTP and/or EFV, we considered a model in which RT interacted with the fluorescein dye attached to the 5'-end of the DNA template. Solving for Equation 5 by using an *r* value of 0.086 (*r* for the RT-T/P-TTP complex), θ_{slow} of 44.8 ns (calculated for the RT-T/P complex; see Methods) and θ_{fast} of 0.37 ns (the empirically determined value for free fluorescein; (17)), we determined that α_{slow} and α_{fast} were 0.17 and 0.83, respectively. Therefore, 83% of the observed *r* value of the RT-T/P-dNTP complex can be attributed to fast dye tumbling. As such, fluorescein is relatively free to rotate when the enzyme forms a polymerase competent RT-T/P-dNTP complex. This observation is different from that of Cy3, which directly interacts with RT when it forms the ternary complex (Supplementary Figure 6a). However, the fluorescein linker (~ 2.5 nm) is significantly longer than the Cy3 linker (~ 0.5 nm) and therefore the fluorescein dye is free to rotate independently of the RT-T/P-dNTP complex. In light of this, we considered a model in which RT can intermittently interact with the terminal fluorescein as it slides along the T/P substrate, increasing the value of α_{slow} by restricting the rotation of the fluorescein and committing the dye to the slow rotation of the RT-T/P complex. In our model, the origin of RT is defined as the polymerase competent ternary complex, centered on the 19 base pair duplex portion of the T/P (see Supplementary Figure 1c). We considered the T/P as a simple linear lattice model with 1 base pair increments and we assumed that RT can slide up to 10 base pairs in either direction. As RT crosses the threshold where it can interact with fluorescein, the value of α_{slow} becomes 1, with the assumption that RT completely confines the local rotation of fluorescein. We next tested the predicted response of anisotropy in our model to changes in the relative distribution of RT on the T/P substrate by employing a simple normal probability distribution function centered on the T/P origin,

$$f(x_{T/P}) = \frac{1}{\sigma\sqrt{2\pi}}\, e^{\frac{-(x_{T/P}-\mu)^2}{2\sigma^2}} \qquad (8)$$

where $x_{T/P}$ is the distance from the origin (-10 base pairs to 10 base pairs (including 0) in increments of 1 base pair), μ is the mean or origin position at 0, and σ is the standard deviation of RT on the T/P in base pairs. The assumption of a normal distribution was used for simplicity,

although we note that many other probability distribution functions could also work in our model. To calculate the predicted anisotropy at each T/P lattice point, we used a modified version of Equation 5,

$$r_{T/P}=\alpha_{slow}\cdot r_o/(1+\tau_{slow}/\gamma\cdot\theta_{slow}) + \alpha_{fast}\cdot r_o/(1+\tau_{fast}/\theta_{fast}) \quad (9)$$

where α_{slow} and α_{slow} are dictated by the model in Supplementary Figure 1c for every T/P position, and γ is a variable scaling constant for θ_{slow} ($1 \leq \gamma \leq 10$) used to quantify the effect of changes in hydrodynamic radius and rotational correlation time on the expected r_{model} value. Finally, to calculate the response of anisotropy to σ, the width of the distribution of RT, we calculated the expected r value (r_{model}) for a range of σ values, by simply multiplying $r_{T/P}$, the r value predicted for each T/P lattice point while RT is centered on that point, by $f(x_{T/P})$, the fractional probability of the center of RT occupying that lattice point:

$$r_{model} = \sum_{x_{T/P}=-10}^{10} f(x_{T/P}) \cdot r_{T/P} \Bigg|_{0\leq\sigma\leq 6} \quad (10)$$

where the expression for r_{model} was evaluated for the indicated range of σ values (see Supplementary Figure 1d; arrow indicates increase in γ). To calculate values of σ predicted to yield the experimentally observed r values for the RT-T/P, RT-TP-TTP, and RT-T/P-EFV complexes under saturating concentrations of RT, we matched experimentally derived r values to those derived from the model and extrapolated to obtain σ (see Supplementary Figure 1D, dotted lines). We then used the predicted values of σ to model theoretical continuous normal probability distributions of RT on the T/P substrate in order to approximate the response of the RT distribution to either dNTP or EFV (Figure 1B).

Cy3 parameters used in AMD simulations

The unmodified Cy3 linker dihedral parameters (see Supplementary Methods) are provided below. The remaining Cy3 parameters can be found in the supplementary information of Spiriti *et al* (15).

```
CG2R52 CG2DC2 CG2DC2 CG2DC2     0.5600  1   180.00
CG2R52 CG2DC2 CG2DC2 CG2DC2     3.3000  2   180.00
NG2R52 CG2R52 CG2DC2 CG2DC2     0.5600  1   180.00
NG2R52 CG2R52 CG2DC2 CG2DC2     3.3000  2   180.00
CG2DC2 CG2DC2 CG2DC2 HGA4       2.5000  2   180.00
CG2R52 CG2DC2 CG2DC2 HGA4       2.5000  2   180.00
```

Supplementary Discussion

Regarding our proposed mechanism of EFV resistance by K103N, it is interesting that K103N is by far the most selected EFV-resistant variant at this codon, since other amino acid replacements would be predicted to similarly destabilize the E138-K101 salt bridge. However, other substitutions at codon K103 in RT (i.e., T/Q/H) that confer resistance to EFV and other NNRTIs (Harrigan et al., 2005) have only rarely been observed in patient isolates; our goal here was to discover and confirm the mechanism for the best-characterized NNRTI and mutant as the platform for future study of additional inhibitors and mutants.

Further, other known NNRTI resistance mutations target the E138-K101 salt bridge that confer moderate resistance to NVP and EFV, most notably K101E and E138K (19, 20), yet the importance of this residue pair on RT conformational dynamics was not previously known. In the structures of NVP-bound K101E and E138K RT (21), the respective E138-E101 O-O and K138-K101 Nζ-Nζ distances (7.4 Å and 5.9 Å) are relatively close, demonstrating that NVP (and likely EFV) is able to force this p51/p66 interface together despite proximal similar charges. Further, in the WT RT-EFV crystal structure (22), the K101 side chain is in register with that of K103. We postulate that K103N allows the side chain of K101 to swing out of the salt-bridging geometry with E138, destabilizing this critical interaction. Thus, it is possible that the K103N mutation relieves a significant steric barrier for K101 that remains intact in the presence of the E138 or K101 mutants. These observations support the previous hypothesis that NNRTIs act as a

“molecular wedge” (23) in this critical hinge region, with our data indicating that the E138-K101 salt bridge stabilizes these interactions to support an open RT conformation.

In regard to the role of the salt bridge in K103N mechanism, our FRET data agrees with the crystallographic observation that K103N-EFV retains the E138-K101R salt bridge (24), since the thumb/fingers distance of K103N RT was essentially the same as that of WT RT. Our data additionally indicates a closed thumb/fingers conformation only in the presence of dNTP, indicating that, in some scenarios (such as K103N) dNTP binding can counteract the allosteric effects of NNRTI on the thumb/fingers distance. If our model of EFV resistance by K103N is correct, we would therefore predict that a crystal structure of the K103N RT-T/P-EFV-dNTP complex would show a broken E138-K101 salt bridge. Further, it is interesting that NVP induced less RT mobility than EFV, and the E138D mutation had little effect on NVP susceptibility even though the E138-K101 salt bridge appears in the WT-NVP structure (25). Combined with the observation that RT displays a closed thumb/fingers conformation in the presence of both NVP and dNTP, suggesting that NVP cannot bind the ternary RT-T/P-dNTP complex (consistent with recent ITC data (26)), we hypothesize that the degree to which NVP forces the E138-K101 salt bridge is less than that of EFV, accounting for its relatively lower NNRTI activity, perhaps owed in part to its molecular structure in the binding pocket which is unable to hold E138 in place for optimal salt bridge geometry. Since our data indicate an allosteric ‘tug-of-war’ between NNRTI and dNTPs which directs the thumb/fingers grip of RT, even a small increase in electrostatic energy in the salt bridge could account for an increased tendency to bind dNTP. Together with the observation that K103N allows an EFV-bound enzyme to bind dNTP, we therefore suggest that an NNRTI which affords additional energetic stabilization near the p51/p66 hinge domain (including additional stabilization of the salt bridge and surrounding residues) would potentially inhibit RT with increased potency over current NNRTIs.

Supplementary References

1. Lansdon,E.B., Brendza,K.M., Hung,M., Wang,R., Mukund,S., Jin,D., Birkus,G., Kutty,N. and Liu,X. (2010) Crystal structures of HIV-1 reverse transcriptase with etravirine (TMC125) and rilpivirine (TMC278): implications for drug design. *J. Med. Chem.*, **53**, 4295-4299.
2. Le Grice,S.F., Cameron,C.E. and Benkovic,S.J. (1995) Purification and characterization of human immunodeficiency virus type 1 reverse transcriptase. *Methods Enzymol.*, **262**, 130-144.
3. Le Grice,S.F. and Gruninger-Leitch,F. (1990) Rapid purification of homodimer and heterodimer HIV-1 reverse transcriptase by metal chelate affinity chromatography. *Eur. J. Biochem.*, **187**, 307-314.
4. Radzio,J. and Sluis-Cremer,N. (2011) Subunit-specific mutational analysis of residue N348 in HIV-1 reverse transcriptase. *Retrovirology*, **8**, 69.
5. Anderson,B.J., Larkin,C., Guja,K. and Schildbach,J.F. (2008) Using fluorophore-labeled oligonucleotides to measure affinities of protein-DNA interactions. *Methods Enzymol.*, **450**, 253-272.
6. Owen,B. and McMurray,C. (2009) Rapid method for measuring DNA binding to protein using fluorescence anisotropy. *Protocol Exchange*, http://www.nature.com/protocolexchange/protocols/557
7. Lakowicz,J.R. (2006) Principles of Fluorescence Spectroscopy. Springer, New York.
8. Hammer,M., Schweitzer,D., Richter,S. and Konigsdorffer,E. (2005) Sodium fluorescein as a retinal pH indicator? *Physiol. Meas.*, **26**, N9-12. Epub 2005 Apr 15.

9. Scalettar,B.A., Abney,J.R., and Hackenbrock,C.R. (1991) Dynamics, structure, and function are coupled in the mitochondrial matrix. *Proc. Natl. Acad. Sci. USA*, **88**, 8057-8061.

10. Fagerburg,M.V. and Leuba,S.H. (2011) Optimal practices for surface-tethered single molecule total internal reflection fluorescence resonance energy transfer analysis. *Methods Mol. Biol.*, **749**, 273-289.

11. Hinterdorfer,P. and van Oijen,A. (eds.) (2009) Handbook of single-molecule biophysics. Springer, New York.

12. Bronson,J.E., Fei,J., Hofman,J.M., Gonzalez,R.L.,Jr. and Wiggins,C.H. (2009) Learning rates and states from biophysical time series: a Bayesian approach to model selection and single-molecule FRET data. *Biophys. J.*, **97**, 3196-3205.

13. Phillips,J.C., Braun,R., Wang,W., Gumbart,J., Tajkhorshid,E., Villa,E., Chipot,C., Skeel,R.D., Kalé,L. and Schulten,K. (2005) Scalable molecular dynamics with NAMD. *J. Comput. Chem.*, **26**, 1781-1802.

14. Iqbal,A., Wang,L., Thompson,K.C., Lilley,D.M. and Norman,D.G. (2008) The structure of cyanine 5 terminally attached to double-stranded DNA: implications for FRET studies. *Biochemistry*, **47**, 7857-7862.

15. Spiriti,J., Binder,J.K., Levitus,M. and van der Vaart,A. (2011) Cy3-DNA stacking interactions strongly depend on the identity of the terminal basepair. *Biophys. J.*, **100**, 1049-1057.

16. Humphrey,W., Dalke,A. and Schulten,K. (1996) VMD: visual molecular dynamics. *J. Mol. Graph.*, **14**, 33-38, 27-38.

17. Ferguson,B.Q. and Yang,D.C. (1986) Methionyl-tRNA synthetase induced 3'-terminal and delocalized conformational transition in tRNAfMet: steady-state fluorescence of tRNA with a single fluorophore. *Biochemistry*, **25**, 529-539.

18. Harrigan,P.R., Mo,T., Wynhoven,B., Hirsch,J., Brumme,Z., McKenna,P., Pattery,T., Vingerhoets,J. and Bacheler,L.T. (2005) Rare mutations at codon 103 of HIV-1 reverse transcriptase can confer resistance to non-nucleoside reverse transcriptase inhibitors. *AIDS*, **19**, 549-554.

19. Xu,H.T., Colby-Germinario,S.P., Huang,W., Oliveira,M., Han,Y., Quan,Y., Petropoulos,C.J. and Wainberg,M.A. (2013) Role of the K101E substitution in HIV-1 reverse transcriptase in resistance to rilpivirine and other nonnucleoside reverse transcriptase inhibitors. *Antimicrob. Agents Chemother.*, **57**, 5649-5657.

20. Ren,J. and Stammers,D.K. (2008) Structural basis for drug resistance mechanisms for non-nucleoside inhibitors of HIV reverse transcriptase. *Virus Res.*, **134**, 157-170.

21. Ren,J., Nichols,C.E., Stamp,A., Chamberlain,P.P., Ferris,R., Weaver,K.L., Short,S.A. and Stammers,D.K. (2006) Structural insights into mechanisms of non-nucleoside drug resistance for HIV-1 reverse transcriptases mutated at codons 101 or 138. *FEBS J.*, **273**, 3850-3860.

22. Ren,J., Milton,J., Weaver,K.L., Short,S.A., Stuart,D.I. and Stammers,D.K. (2000) Structural basis for the resilience of efavirenz (DMP-266) to drug resistance mutations in HIV-1 reverse transcriptase. *Structure*, **8**, 1089-1094.

23. Ivetac,A. and McCammon,J.A. (2009) Elucidating the inhibition mechanism of HIV-1 non-nucleoside reverse transcriptase inhibitors through multicopy molecular dynamics simulations. *J. Mol. Biol.*, **388**, 644-658.

24. Lindberg, J., Sigurdsson, S., Lowgren, S., Andersson, H.O., Sahlberg, C., Noreen, R., Fridborg, K., Zhang, H., and Unge, T. (2001). Structural basis for the inhibitory efficacy of efavirenz (DMP-266), MSC194 and PNU142721 towards the HIV-1 RT K103N mutant. Eur.J.Biochem. *269*, 1670–1677.

25. Ren,J., Esnouf,R., Garman,E., Somers,D., Ross,C., Kirby,I., Keeling,J., Darby,G., Jones,Y., Stuart,D., et al. (1995) High resolution structures of HIV-1 RT from four RT-inhibitor complexes. *Nat. Struct. Biol.*, **2**, 293-302.

26. Bec,G., Meyer,B., Gerard,M.A., Steger,J., Fauster,K., Wolff,P., Burnouf,D., Micura,R., Dumas,P. and Ennifar,E. (2013) Thermodynamics of HIV-1 reverse transcriptase in action elucidates the mechanism of action of non-nucleoside inhibitors. *J. Am. Chem. Soc.*, **135**, 9743-9752.
